# First-line immunochemotherapy for advanced NSCLC in Asian patients: a meta-analysis of phase 3 RCTs

**DOI:** 10.3389/fonc.2025.1709348

**Published:** 2025-11-19

**Authors:** Zhifang Mao, Zhiyong Zhang, Meijiao Song, Jiaqi He, Jing Zheng, Wenquan Liu

**Affiliations:** 1 Department of Oncology, Jiangxi Provincial People’s Hospital, The First Affiliated Hospital of Nanchang Medical College, Nanchang, China; 2 Department of Clinical Laboratory, Jiangxi Provincial People’s Hospital, The First Affiliated Hospital of Nanchang Medical College, Nanchang, China; 3 Department of Oncology, The Second Affiliated Hospital of Jiujiang University, Jiujiang, China

**Keywords:** PD-1/PD-L1 inhibitors, chemotherapy, Asian, non-small-cell lung cancer, meta-analysis

## Abstract

**Background:**

PD-1/PD-L1 inhibitors plus chemotherapy (PC) are now broadly acknowledged as effective for treating stage IIIb–IV non-small cell lung cancer (NSCLC). However, data specific to Asian populations remain limited, and updated evidence from randomized controlled trials (RCTs) is warranted. In this study, the efficacy and safety of PC are analyzed and compared with those of chemotherapy in this population.

**Methods:**

Six databases were systematically explored to locate applicable phase 3 RCTs. Eligible studies involved Asian patients with stage IIIb–IV NSCLC and compared PC treatment with conventional chemotherapy. Overall survival (OS) and progression-free survival (PFS) were regarded as primary endpoints.

**Results:**

A total of 16 phase 3 RCTs involving 4,452 Asian patients were included. Compared with chemotherapy alone, PC significantly improved OS (HR: 0.68 [0.63, 0.75], *p* < 0.00001, *I*^2^ = 30%) and PFS (HR: 0.50 [0.47, 0.54], *p* < 0.00001, *I*^2^ = 39%). The survival benefits were consistent across most subgroups and increased as survival time increased. The objective response rate (RR: 1.62 [1.51, 1.74], *p* < 0.00001, *I*^2^ = 0%) and disease control rate (RR: 1.09 [1.05, 1.12], *p* < 0.00001, *I*^2^ = 7%) were also better in the PC group. Brain metastasis and a PD-L1 CPS >50% were favorable factors in the PC group. However, more immune-related AEs (irAEs) were found in the PC group.

**Conclusion:**

Among Asian patients with stage IIIb–IV NSCLC, PC therapy still has a notable advantage in prolonging survival. Nonetheless, the increased frequency of AEs, particularly irAEs, warrants close attention.

**Systematic Review Registration:**

https://www.crd.york.ac.uk/PROSPERO/view/CRD420251022604, PROSPERO identifier CRD420251022604.

## Introduction

Approximately 85% of pulmonary malignancies fall under the category of non-small cell lung cancer (NSCLC), with many detected at stage IIIb–IV, where curative therapies remain limited (1). Over the past decade, the application of immune checkpoint inhibitors (ICIs), which block programmed death-1 (PD-1)/programmed death-ligand 1 (PD-L1) signaling, has dramatically reshaped therapeutic approaches to advanced NSCLC (2). These agents, especially in combination with chemotherapy, have shown marked survival benefits over chemotherapy alone, setting a new benchmark for first-line therapy (3).

Multiple studies have demonstrated that PD-1/PD-L1 inhibitors + chemotherapy (PC) is beneficial for stage IIIb–IV NSCLC (4–19). As shown in KEYNOTE-189, pembrolizumab with chemotherapy significantly enhanced progression-free survival (PFS) among non-squamous NSCLC cases, irrespective of PD-L1 levels (13). Similarly, in the KEYNOTE-407 trial, survival was improved when pembrolizumab was administered alongside chemotherapy in individuals with squamous NSCLC (14). The IMpower150 study further supported these findings, revealing that atezolizumab + bevacizumab + chemotherapy improved overall survival (OS) in patients with non-squamous NSCLC (20).

Despite the global success of these combination therapies, the majority of pivotal trials have been conducted in predominantly Western populations, leading to a paucity of data specific to Asian patients (21–23). This is particularly concerning given the distinct genetic, environmental, and epidemiological factors that influence NSCLC in Asian populations. For example, compared to Western populations, EGFR mutations occur more commonly in East Asians, which may influence treatment responses and clinical outcomes (24). Moreover, differences in drug metabolism, tolerability, and adverse event profiles necessitate tailored investigations to optimize therapeutic strategies for Asian patients. Recognizing these disparities, recent studies have begun to focus on the effectiveness and tolerability of PC therapy among Asian populations. In the GEMSTONE-302 trial, compared with the chemotherapy-alone group, PFS and OS were notably better in the PC group, which also showed acceptable safety outcomes (10). The efficacy of sintilimab plus chemotherapy was assessed in another study, revealing favorable outcomes irrespective of PD-L1 expression levels (15).

While these studies provide valuable insights, they are limited by sample size and the heterogeneity of study designs. An updated meta-analysis incorporating data from recent phase 3 randomized controlled trials (RCTs) is essential to derive more robust conclusions concerning how PC therapy compares to chemotherapy alone in terms of efficacy and risk among Asian patients diagnosed with stage IIIb–IV NSCLC. By synthesizing data from multiple high-quality RCTs, this study seeks to deliver thorough evidence to support clinical decision-making and improve therapeutic planning in this population.

## Materials and methods

### Search strategy

Literature up to 15 March 2025 was retrieved from databases such as PubMed, ScienceDirect, Cochrane Library, EMBASE, Scopus, and Web of Science. The search strategy included combinations of the following MeSH terms: “PD-1/PD-L1 inhibitors,” “lung cancer,” and “randomized” (**Supplementary Table S1**).

### Selection criteria

Studies were selected when all of the following conditions were met: 1) were phase 3 RCTs; 2) included individuals diagnosed with stage IIIb–IV NSCLC; 3) either involved Asian participants as a subgroup or focused solely on Asian cohorts. “Asian populations” refers to patients of Asian origin, as defined by the geographical region where the studies were conducted or where subgroup data for Asian patients were explicitly reported; 4) compared the PC group versus chemotherapy alone (chemotherapy group); and 5) included data on at least one measured outcome, such as OS, PFS, response rates, or AEs.

The exclusion criteria were as follows: 1) phase 1/2 design, 2) absence of key efficacy or toxicity endpoints, 3) pooled analyses without specific data for Asian subgroups, or 4) duplicate entries or abstracts lacking complete datasets.

### Data extraction

Data from all included studies were independently collected by two reviewers using a uniform extraction template. Collected variables included baseline demographics (sex, stage, etc.), survival outcomes, response rates, and AEs. Any conflicts were settled through consensus.

### Outcome assessment

OS and PFS subgroup analyses were conducted on the basis of variables including age, sex, race, ECOG PS, smoking status, pathological type, stage, brain metastases, PD-L1 combined positive score (CPS), PD-1/PD-L1 inhibitor type, and platinum chemotherapy type.

### Quality assessment

Two independent reviewers evaluated trial quality and potential bias with the Cochrane tool and Jadad scale. Jadad scoring is based on a 7-point system, where a score between 4 and 7 denotes high study quality (25, 26). Furthermore, evidence strength was graded via the GRADE approach as high, moderate, low, or very low (27).

### Statistical analysis

Statistical analyses were conducted using RevMan version 5.4 and STATA 17.0 software. Hazard ratios (HRs) and risk ratios (RRs) were applied to synthesize time-to-event outcomes and binary data from included studies. Between-study heterogeneity was assessed via Cochrane’s Q and *I*² statistics; *I*² over 50% or *p <*0.10 indicated notable heterogeneity. When heterogeneity was substantial, a random-effects model was used; a fixed-effects model was selected under low heterogeneity conditions. Publication bias was examined using funnel diagrams, along with Egger’s and Begg’s statistical tests (28–30). To assess robustness, sensitivity analysis excluded studies one at a time. A two-sided *p <*0.05 was considered statistically significant.

## Results

### Search results

**Figure 1:**
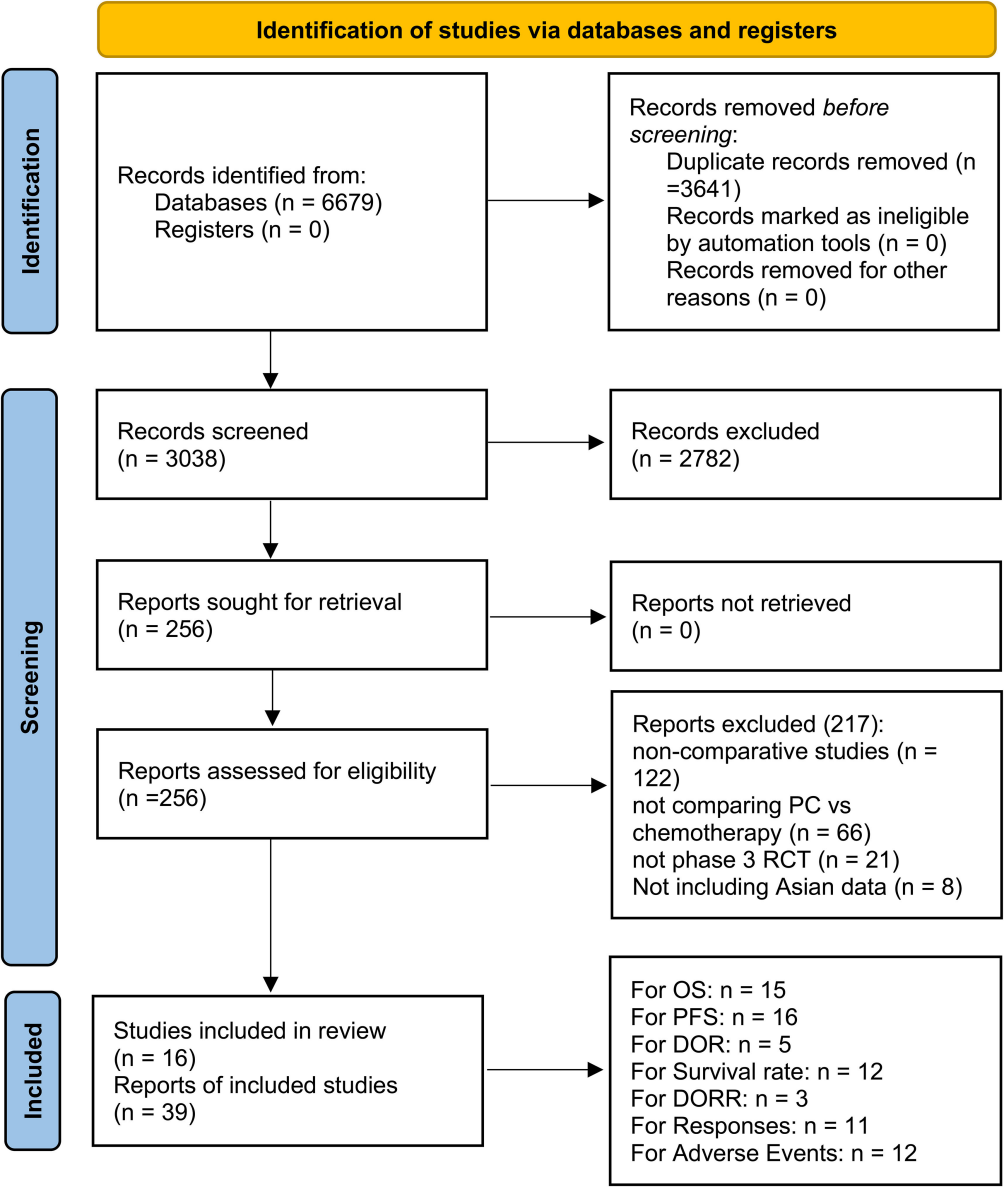
Flowchart.

Ultimately, this meta-analysis incorporated 39 publications derived from 16 phase 3 RCTs, encompassing 4,452 Asian individuals diagnosed with stage IIIb–IV NSCLC (4–19, 31–53). **Figure 1** displays the study selection procedure in a flow diagram formatted according to the PRISMA 2020 standards. Nine RCTs were conducted exclusively in Asia (4, 6–8, 10, 15, 16, 18, 19). The other seven RCTs involved global multicenter designs, with analyses performed on Asian subgroup data (5, 9, 11–14, 17). Three RCTs were subjected to subgroup analysis separately on the basis of the countries where the included patients were located (12–14). The baseline characteristics are detailed in **Table 1**. Overall study quality was rated as high across all included trials (**Supplementary Figure S1**, **Supplementary Table S2**). The evidence levels, assessed via the GRADE method, ranged between moderate and high (**Supplementary Table S3**).

**Table 1:** Baseline characteristics of the included studies.

Study	Registration no.	Period	Country	Groups	Patients	Sex (M/F)	Age (mean, years)	Pathological type		Stage		PD-1/PD-L1 type	Follow-up (months)
								Sq	Non-sq	III	IV		
AK105-302 (4)	NCT03866993	2018.12–2020.10	China	PC	175	162/13	60.9	175	0	23	152	Penpulimab	24.7
				Chemotherapy	175	162/13	61.9	175	0	26	149
ASTRUM-004 (5)	NCT04033354	2019.08–2021.02	Global multicenter-Asian subgroup	PC	240	–	–	–	–	–	–	Sugemalimab	31.1
				Chemotherapy	119	–	–	–	–	–	–
CameL (6, 31, 32)	NCT03134872	2017.05–2018.06	China	PC	205	146/59	59	0	205	30	175	Camrelizumab	65.2
				Chemotherapy	207	149/58	61	0	207	41	166
CameL-Sq (7)	NCT03668496	2018.11–2019.12	China	PC	193	179/14	64	193	0	54	139	Camrelizumab	13.5
				Chemotherapy	196	180/16	62	197	0	55	141	11.6
CHOICE-01 (8, 33)	NCT03856411	2019.04–2020.08	China	PC	309	247/62	63	147	162	49	260	Toripalimab	21.2
				Chemotherapy	156	130/26	61	73	83	23	133
EMPOWER-Lung 3 (9, 34, 35)	NCT03409614	2019.06–2020.09	Global multicenter-Asian subgroup	PC	42	–	–	–	–	–	–	Cemiplimab	28.4
				Chemotherapy	16	–	–	–	–	–	–
GEMSTONE-302 (10, 36)	NCT03789604	2018.12–2020.03	China	PC	320	254/66	62	129	191	0	320	Sugemalimab	25.6
				Chemotherapy	159	129/30	64	63	96	0	159
IMpower131 (11)	NCT02367794	2015.06–2017.03	Global multicenter-Asian subgroup	PC	41	–	–	–	–	–	–	Atezolizumab	18.1
				Chemotherapy	37	–	–	–	–	–	–	16.1
IMpower132 (12, 37, 38)	NCT02657434	2016.04–2017.03	Global multicenter-Asian subgroup	PC	71	–	–	–	–	–	–	Atezolizumab	14.8
				Chemotherapy	65	–	–	–	–	–	–
KEYNOTE-189 (13, 39–43)	NCT02578680	2016.02–2017.03	Global multicenter-Asian subgroup	PC	25	–	–	–	–	–	–	Pembrolizumab	64.6
				Chemotherapy	15	–	–	–	–	–	–
KEYNOTE-407 (14, 44–48)	NCT02775435	2016.08–2017.12	Global multicenter-Asian subgroup	PC	54	–	–	–	–	–	–	Pembrolizumab	56.9
				Chemotherapy	52	–	–	–	–	–	–
ORIENT-11 (15, 49, 50)	NCT03607539	2018.08–2019.07	China	PC	266	204/62	61	0	266	21	245	Sintilimab	30.8
				Chemotherapy	131	99/32	61	0	131	15	116
ORIENT-12 (16)	NCT03629925	2018.08–2019.07	China	PC	179	163/16	64	179	0	39	140	Sintilimab	8.0
				Chemotherapy	178	164/14	62	178	0	44	134
POSEIDON (17, 51)	NCT03164616	2017.06–2018.09	Global multicenter-Asian subgroup	PC	123	–	–	–	–	–	–	Durvalumab	63.4
				Chemotherapy	128	–	–	–	–	–	–
RATIONALE-304 (18, 52)	NCT03663205	2018.07–2019.07	China	PC	223	168/55	60	0	223	40	183	Tislelizumab	16.1
				Chemotherapy	111	79/32	61	0	111	24	87
RATIONALE-307 (19, 53)	NCT03594747	2018.07–2019.06	China	PC	120	107/13	60	120	0	38	82	Tislelizumab	20.5
				Chemotherapy	121	111/10	62	121	0	44	77

Three RCTs (IMpower132, KEYNOTE-189, and KEYNOTE-407) were subjected to subgroup analysis separately based on the countries where the included patients were located.

M/F, male/female; PC, PD-1/PD-L1 inhibitors combined with chemotherapy; PD-1, programmed death-1; PD-L1, programmed death-ligand 1.

### Survival

**Figure 2:**
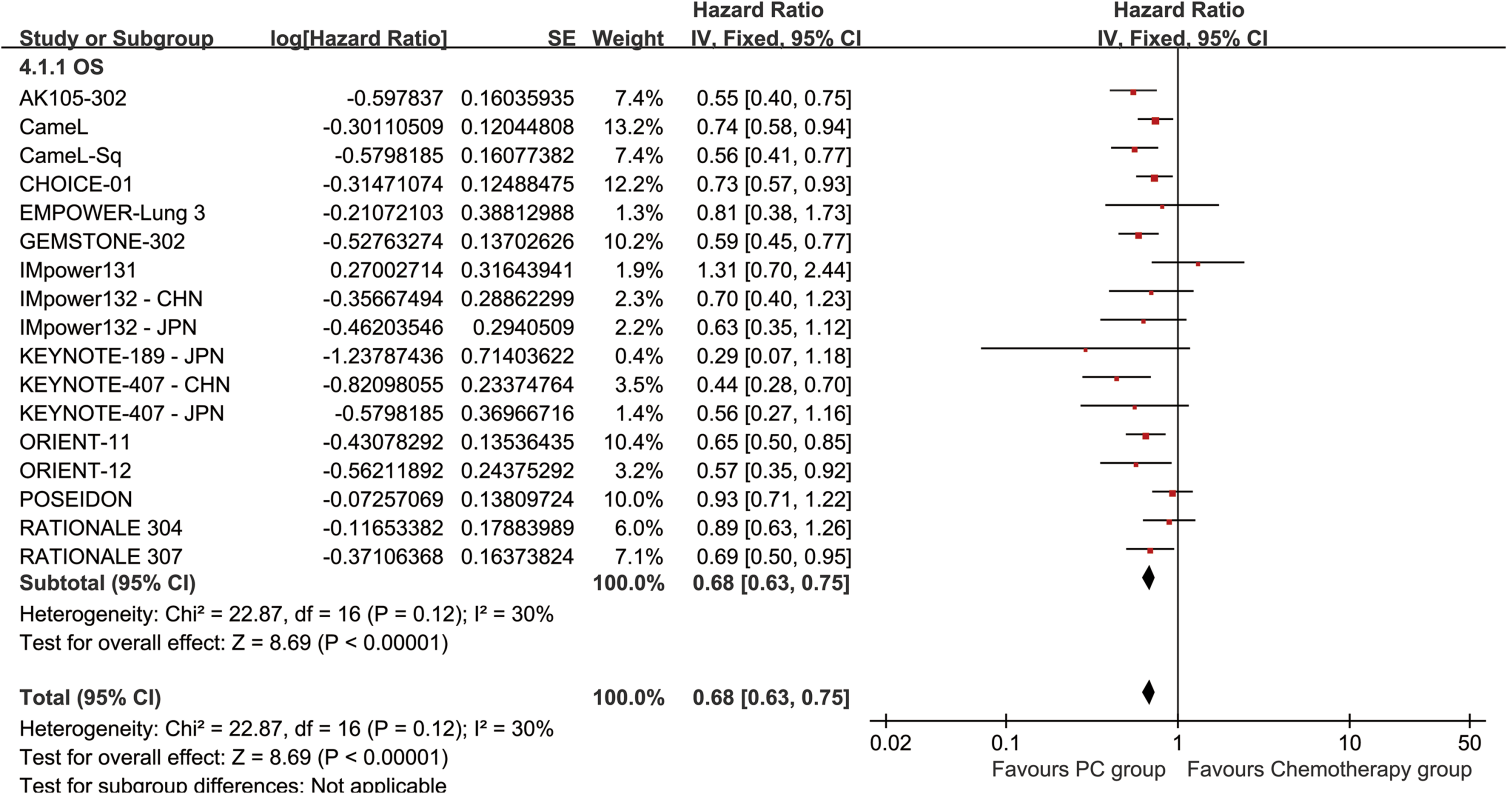
Forest plot of overall survival associated with PC versus chemotherapy.

**Figure 3:**
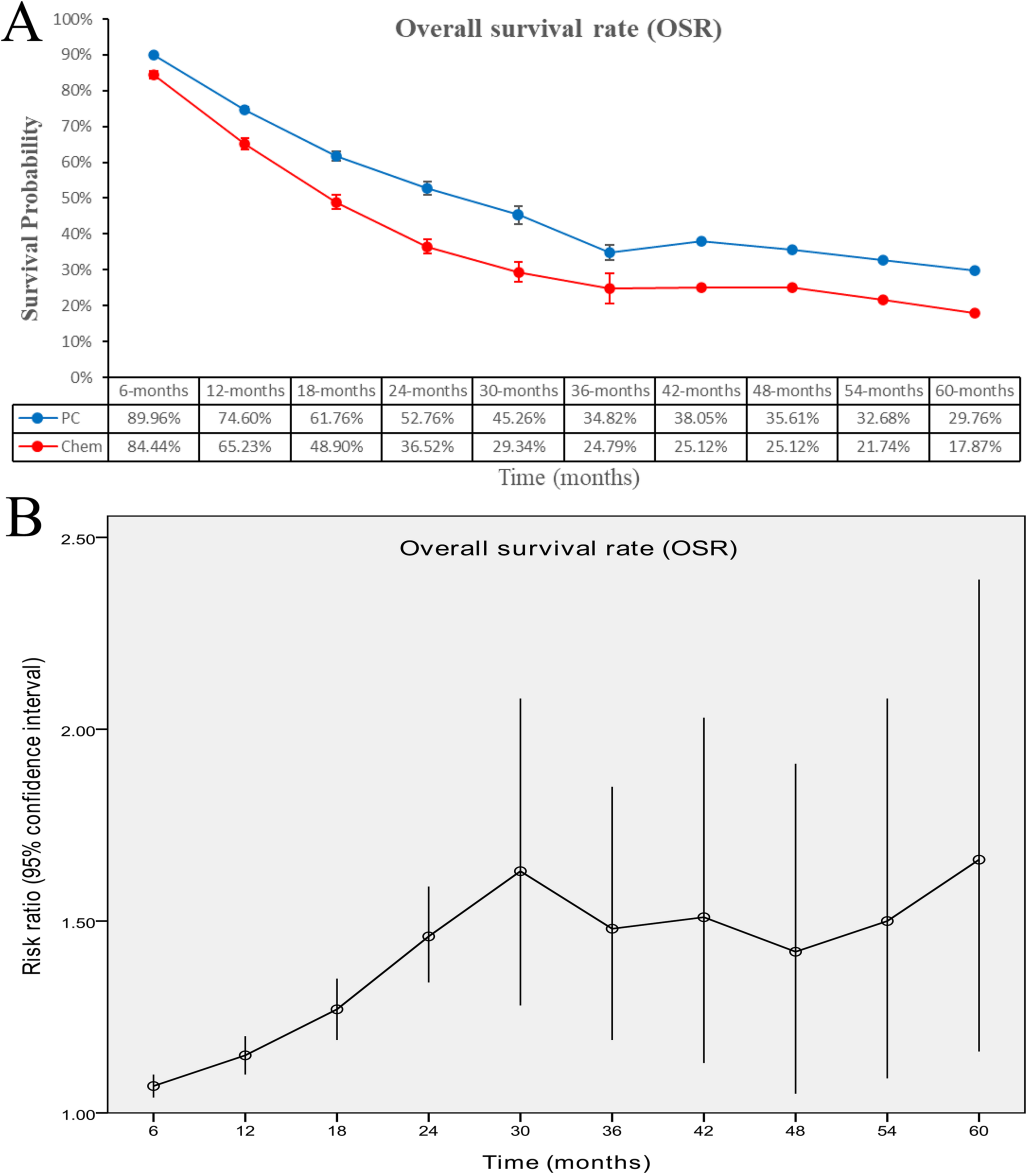
Comparisons of OSR associated with PC versus chemotherapy. **(A)** OSR at 6–60 months; **(B)** trend of risk ratios in OSR.

PC therapy showed significantly superior OS (HR: 0.68 [0.63, 0.75], *p* < 0.00001, *I*^2^ = 30%) (**Figure 2**). The overall survival rate (OSR) was significantly greater for the PC group over a period of 6–60 months (3-year OSR: 34.82% vs. 24.79%; 5-year OSR: 29.76% vs. 17.87%) (**Figure 3**, **Supplementary Figure S2**).

**Figure 4:**
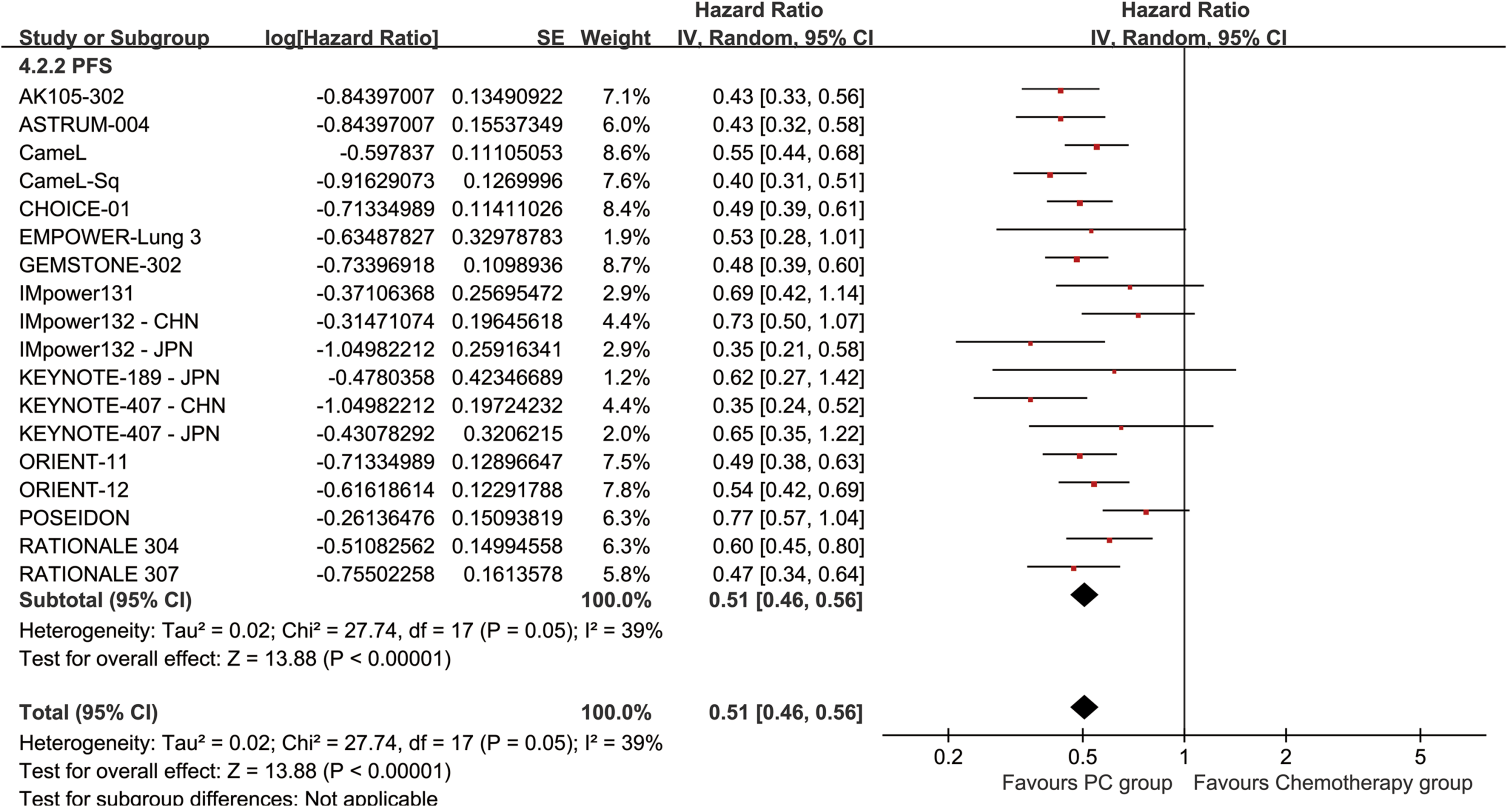
Forest plot of progression-free survival associated with PC versus chemotherapy.

**Figure 5:**
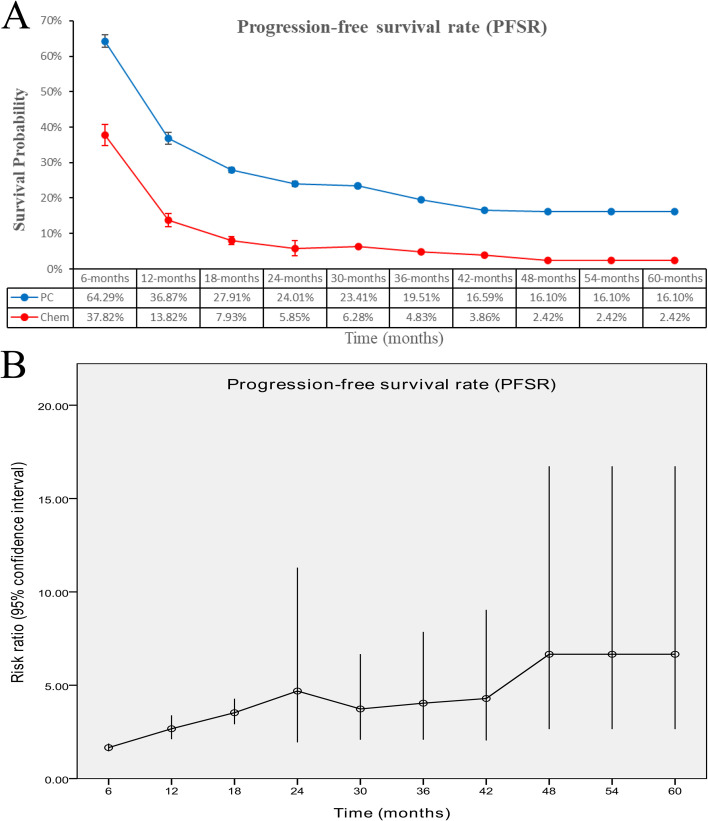
Comparisons of PFSR associated with PC versus chemotherapy. **(A)** PFSR at 6–60 months; **(B)** trend of risk ratios in PFSR.

The PC group had increased PFS (HR: 0.50 [0.47, 0.54], *p* < 0.00001, *I*^2^ = 39%) (**Figure 4**). Progression-free survival rate (PFSR) displayed a significant advantage for the PC group over a duration of 6–60 months (3-year PFSR: 19.51% vs. 4.83%; 5-year PFSR: 16.10% vs. 2.42%) (**Figure 5**, **Supplementary Figure S3**).

### Subgroup analysis

Across various baseline characteristics, PC treatment showed uniform survival advantages in OS and PFS, as detailed in the outcome section. Brain metastasis and a PD-L1 CPS >50% predicted improved OS and PFS outcomes among patients receiving PC (**Table 2**).

**Table 2:** Subgroup analysis of overall survival and progression-free survival.

Subgroups	Overall survival				Progression-free survival			
	Included studies	Patients	HR [95% CI]	*p*	Included studies	Patients	HR [95% CI]	*p*
Total	15	4,293	0.68 [0.63, 0.75]	<0.00001	16	4,452	0.50 [0.47, 0.54]	<0.00001
Age								
<65 years	6	1,479	0.64 [0.56, 0.74]	<0.00001	9	2,082	0.46 [0.41, 0.51]	<0.00001
>65 years	6	868	0.78 [0.65, 0.93]	0.006	9	1,342	0.55 [0.48, 0.62]	<0.00001
Sex								
Female	5	416	0.75 [0.56, 1.00]	0.05	7	565	0.65 [0.53, 0.80]	<0.0001
Male	5	1,581	0.69 [0.61, 0.79]	<0.00001	8	2,509	0.48 [0.43, 0.52]	<0.00001
ECOG PS								
0	6	542	0.62 [0.48, 0.80]	0.0002	9	739	0.51 [0.42, 0.61]	<0.00001
1	6	1,805	0.71 [0.62, 0.80]	<0.00001	9	2,685	0.49 [0.45, 0.54]	<0.00001
Smoking status								
Current/former	5	1,443	0.60 [0.52, 0.70]	<0.00001	8	2,290	0.45 [0.40, 0.50]	<0.00001
Never	5	492	0.88 [0.67, 1.16]	0.36	8	722	0.59 [0.49, 0.70]	<0.00001
Pathological type								
Squamous	9	2,042	0.64 [0.52, 0.80]	<0.0001	10	2,361	0.45 [0.41, 0.50]	<0.00001
Non-squamous	8	1,979	0.68 [0.60, 0.77]	<0.00001	8	1,979	0.54 [0.49, 0.61]	<0.00001
Stage								
III	5	327	0.78 [0.55, 1.11]	0.17	7	492	0.43 [0.34, 0.54]	<0.00001
IV	12	2,895	0.66 [0.59, 0.73]	<0.00001	13	3,328	0.51 [0.47, 0.55]	<0.00001
Brain metastases								
Yes	2	75	0.54 [0.29, 1.03]	0.06	3	142	0.38 [0.24, 0.58]	<0.0001
No	2	734	0.68 [0.57, 0.81]	<0.0001	3	1,144	0.53 [0.46, 0.61]	<0.00001
PD-L1 CPS								
<1%	6	817	0.80 [0.67, 0.96]	0.02	9	1,231	0.61 [0.53, 0.70]	<0.00001
>1%	5	1,134	0.59 [0.50, 0.70]	<0.00001	8	1,804	0.44 [0.40, 0.50]	<0.00001
1%–49%	5	764	0.70 [0.57, 0.85]	0.0003	8	1,079	0.54 [0.47, 0.63]	<0.00001
>50%	5	405	0.54 [0.39, 0.74]	0.0002	8	760	0.40 [0.33, 0.49]	<0.00001
PD-1/PD-L1 inhibitor type								
Penpulimab	1	350	0.55 [0.40, 0.75]	0.0002	1	350	0.43 [0.33, 0.56]	<0.00001
Sugemalimab	1	479	0.59 [0.45, 0.77]	0.0001	2	838	0.46 [0.39, 0.55]	<0.00001
Camrelizumab	2	801	0.67 [0.55, 0.81]	<0.0001	2	801	0.47 [0.35, 0.64]	<0.00001
Toripalimab	1	465	0.73 [0.57, 0.93]	0.10	1	465	0.49 [0.39, 0.61]	<0.00001
Cemiplimab	1	61	0.81 [0.38, 1.73]	0.59	1	61	0.53 [0.28, 1.01]	0.05
Atezolizumab	3	342	0.81 [0.58, 1.14]	0.23	3	342	0.57 [0.36, 0.89]	0.01
Pembrolizumab	3	215	0.46 [0.31, 0.66]	<0.0001	3	215	0.44 [0.32, 0.60]	<0.00001
Sintilimab	2	794	0.63 [0.50, 0.79]	<0.0001	2	754	0.52 [0.43, 0.61]	<0.00001
Durvalumab	1	251	0.93 [0.71, 1.22]	0.60	1	251	0.77 [0.57, 1.04]	0.08
Tislelizumab	2	575	0.77 [0.61, 0.98]	0.03	2	572	0.54 [0.43, 0.66]	<0.00001
Platinum chemotherapy type								
Cisplatin	1	104	0.53 [0.32, 0.87]	0.01	2	239	0.55 [0.41, 0.73]	<0.0001
Carboplatin	12	2,941	0.67 [0.60, 0.74]	<0.00001	13	3,522	0.48 [0.44, 0.52]	<0.00001

CI, confidence interval; CPS, combined positive score; ECOG PS, Eastern Cooperative Oncology Group Performance Status; HR, hazard ratio; OS, overall survival; PC, PD-1/PD-L1 inhibitors combined with chemotherapy; PD, progressive disease; PD-1, programmed cell death protein 1; PD-L1, programmed death-ligand 1; PFS, progression-free survival.

### Responses

When compared to chemotherapy, PC significantly improved the objective response rate (ORR) (RR: 1.62 [1.51, 1.74], *p* < 0.00001, *I*^2^ = 0%) and the disease control rate (DCR) (RR: 1.09 [1.05, 1.12], *p* < 0.00001, *I*^2^ = 7%) (**Supplementary Figure S4**, **Table 3**).

**Table 3:** Tumor responses.

Responses	PC		Chemotherapy		Risk ratio [95% CI]	*p*
	Event/total	%	Event/total	%		
ORR	1,362/2,232	61.02%	631/1,671	37.76%	1.62 [1.51, 1.74]	<0.00001
DCR	1,694/1,912	88.60%	1,231/1,512	81.42%	1.09 [1.05, 1.12]	<0.00001
CR	45/1,912	2.35%	11/1,512	0.73%	3.21 [1.78, 5.78]	<0.0001
PR	1,114/1,912	58.26%	558/1,512	36.90%	1.58 [1.46, 1.70]	<0.00001
SD	536/1,912	28.03%	662/1,512	43.78%	0.61 [0.52, 0.72]	<0.00001
PD	136/1,912	7.11%	199/1,512	13.16%	0.55 [0.45, 0.68]	<0.00001

CI, confidence interval; CR, complete response; DCR, disease control rate; ORR, objective response rate; PC, PD-1/PD-L1 inhibitors combined with chemotherapy; PD, progressive disease; PD-1, programmed cell death protein 1; PD-L1, programmed death-ligand 1; PR, partial response; RR, risk ratio; SD, stable disease.

**Figure 6:**
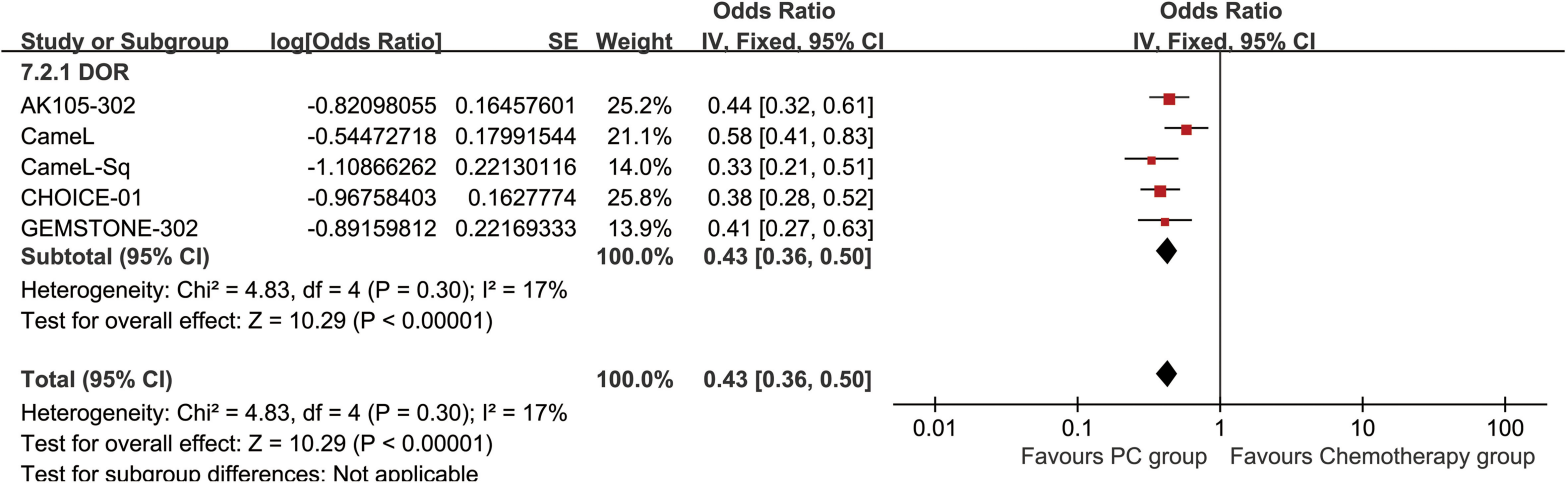
Forest plot of duration of response associated with PC versus chemotherapy.

**Figure 7:**
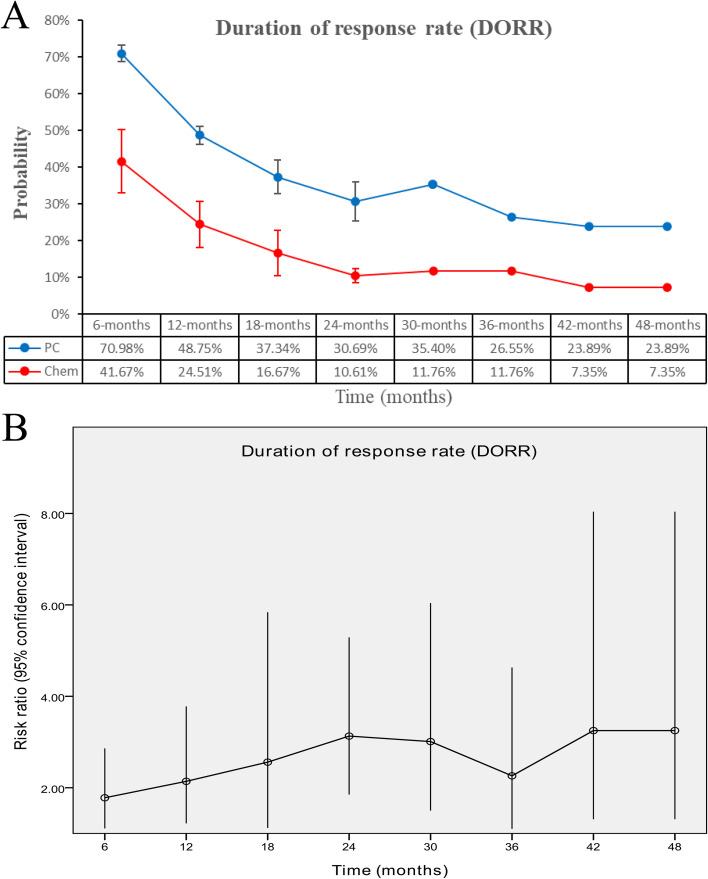
Comparisons of DORR associated with PC versus chemotherapy. **(A)** DORR at 6–48 months; **(B)** trend of risk ratios in DORR.

The PC group had a significantly extended duration of response (DOR) (HR: 0.43 [0.36, 0.50], *p* < 0.00001, *I*^2^ = 17%) (**Figure 6**). Additionally, DOR rates (DORR) favored the PC group at 6–48 months (**Figure 7**, **Supplementary Figure S5**).

### Safety

Overall, the PC group had greater incidences of total treatment-emergent adverse events (TEAEs)/immune-related adverse events (irAEs), grade 3–5 TEAEs/treatment-related adverse events (TRAEs)/irAEs, serious TEAEs/TRAEs/irAEs, and TEAEs/TRAEs leading to discontinuation (**Table 4**).

**Table 4:** Summary of adverse events.

Adverse events	PC		Chemotherapy		Risk ratio [95% CI]	*P*
	Event/total	%	Event/total	%		
TEAEs						
Total TEAEs	2,220/2,232	99.46%	1,650/1,671	98.74%	1.01 [1.00, 1.01]	0.04
Grade 3–5 TEAEs	1,615/2,232	72.36%	1,117/1,671	66.85%	1.10 [1.03, 1.18]	0.005
Serious TEAEs	639/1,675	38.15%	316/1,259	25.10%	1.54 [1.24, 1.91]	<0.0001
TEAEs leading to discontinuation	343/2,232	15.37%	154/1,671	9.22%	1.66 [1.21, 2.27]	0.002
TEAEs leading to death	129/2,232	5.78%	104/1,671	6.22%	1.01 [0.78, 1.30]	0.96
TRAEs						
Total TRAEs	1,219/1,230	99.11%	1,053/1,080	97.50%	1.01 [1.00, 1.03]	0.14
Grade 3–5 TRAEs	825/1,208	68.29%	655/1,052	62.26%	1.13 [1.01, 1.26]	0.04
Serious TRAEs	305/1,023	29.81%	172/871	19.75%	1.57 [1.14, 2.17]	0.006
TRAEs leading to discontinuation	96/958	10.02%	31/797	3.89%	2.26 [1.53, 3.32]	<0.0001
TRAEs leading to death	37/1,110	3.33%	23/959	2.40%	1.44 [0.86, 2.44]	0.17
irAEs						
Total irAEs	760/1,812	41.94%	248/1,240	20.00%	2.53 [1.72, 3.73]	<0.00001
Grade 3–5 irAEs	148/1,764	8.39%	32/1,187	2.70%	2.69 [1.41, 5.14]	0.003
Serious irAEs	11/175	6.29%	2/175	1.14%	5.50 [1.24, 24.45]	0.03
irAEs leading to discontinuation	6/175	3.43%	1/175	0.57%	6.00 [0.73, 49.32]	0.10

AE, adverse event; CI, confidence interval; irAE, immune-related adverse event; PC, PD-1/PD-L1 inhibitors combined with chemotherapy; PD-1, programmed cell death protein 1; PD-L1, programmed death-ligand 1; RR, risk ratio; TEAE, treatment-emergent adverse event; TRAE, treatment-related adverse event.

In the TEAE analysis, the PC group exhibited higher rates of any-grade ALT and AST increased, along with 18 additional TEAEs (**Supplementary Table S4**). Moreover, the PC group also presented higher rates of grade 3–5 lymphocyte count, diarrhea, and rash (**Supplementary Table S5**).

Within the irAE assessment, patients receiving PC had more frequent hypothyroidism cases and seven additional irAEs (**Supplementary Table S6**). Moreover, the PC group also had higher rates of grade 3–5 pneumonitis, severe skin reactions, and hypothyroidism (**Supplementary Table S7**). The top 5 grade 3–5 irAEs in the PC group were pneumonitis (1.81%), severe skin reactions (1.69%), hypothyroidism (1.63%), pneumonia (1.45%), and hepatitis (1.21%).

### Sensitivity analysis

Robustness of results was confirmed as sensitivity analyses showed stable pooled RRs for PFSR-6m, stable disease (SD), and grade 3–5 TEAEs. The exclusion of individual studies did not meaningfully affect effect size or variability, indicating robustness of the overall findings (**Supplementary Figure S6**).

### Publication bias

Visual inspection of funnel plots for OS, PFS, ORR, and TEAEs revealed general symmetry (**Supplementary Figure S7**). Furthermore, no significant publication bias was found by Egger’s and Begg’s tests for these outcomes (all *p* > 0.05) (**Supplementary Figure S8**).

## Discussion

Compared with standard chemotherapy, the introduction of ICIs, especially those that act on the PD-1/PD-L1 axis, has transformed NSCLC therapy by providing notable survival advantages. However, the majority of clinical trials evaluating these therapies have focused predominantly on Western populations, leaving a gap in understanding their efficacy and safety in Asian patients (21–23). Given the distinct genetic, environmental, and epidemiological factors influencing NSCLC in Asian populations, assessing the applicability of these treatments within this demographic is imperative. To bridge this clinical gap, we conducted a meta-analysis comparing PC therapy with chemotherapy alone in Asian individuals with stage IIIb–IV NSCLC. The results indicated that, compared with chemotherapy alone, PC treatment led to notable improvements in OS and PFS. These survival benefits were consistent across various subgroups. Additionally, the PC regimen was associated with a higher ORR and DCR.

Our findings show that the PC regimen leads to a marked decline in both mortality (HR: 0.68) and disease progression (HR: 0.50). Compared with those in global trial populations, the survival benefits of PC therapy appear to be slightly greater in Asian patients. For example, in ASTRUM-004, which included a predominantly non-Asian cohort, the HR for PFS was 0.77, whereas in Asian patients, the PFS HR was 0.42 (5). Ethnic variations in tumor biology may partly explain the enhanced efficacy of PC therapy in Asian patients. Compared with non-Asian populations, Asian NSCLC patients exhibit a higher prevalence of EGFR mutations, distinct immune-related gene expression profiles, and specific gut microbiome compositions that can modulate the tumor immune microenvironment (54). These biological characteristics may contribute to greater immunogenicity and improved synergy between PD-1/PD-L1 inhibitors and chemotherapy. Moreover, pharmacogenomic differences influencing drug metabolism and immune activation could further enhance treatment sensitivity. Notably, long-term survival benefits became more apparent as the follow-up duration increased. This finding is in line with emerging data emphasizing the durability of immunotherapy-driven responses (10, 15). Furthermore, subgroup analyses revealed that the benefit of PC therapy extended to patients traditionally considered at increased risk. Intriguingly, in our subgroup analysis, patients with brain metastases had unexpectedly favorable OS (HR: 0.54) and an even greater improvement in PFS (HR: 0.38). This finding reinforces the growing body of evidence indicating that ICIs, either alone or in combination, possess intracranial activity (54). Additionally, individuals presenting elevated PD-L1 levels (CPS > 50%) had the greatest improvement in OS (HR: 0.54), which supports the biological basis for employing PD-L1 status as a predictive biomarker. However, our study found that patients with PD-L1 CPS under 1% also showed survival benefit (HR 0.80), indicating that PD-L1 status should not be the sole determinant in clinical decision-making. Potential sources of heterogeneity may include variations in PD-L1 testing assays and thresholds across trials, differences in chemotherapy backbones (platinum-doublet regimens such as cisplatin vs. carboplatin), and minor regional variations in trial conduct or patient enrollment criteria among East Asian populations.

Moreover, PC therapy also significantly improved tumor response outcomes (ORR, DCR, CR, and PR) in Asian patients. These findings reflect enhanced tumor shrinkage and control in the PC group. The depth and durability of response are key indicators of therapeutic efficacy. Our analysis also revealed that DOR was significantly prolonged in the PC group (HR: 0.43), with DORR superiority persisting through 6–48 months. These durable responses may be attributed to the sustained immunological pressure exerted by PD-1/PD-L1 blockade, which enhances the antitumor T-cell activity of patients (55). Several individual trials included in our analysis support these observations. In the Chinese CameL study, camrelizumab plus chemotherapy led to a response rate of 60.5%, which was notably higher than that of chemotherapy alone. Similarly, in the RATIONALE-307 study, tislelizumab-based therapy yielded ORRs above 60%, even in PD-L1-low expressers (19). These consistent patterns reinforce the notion that ICIs enhance chemotherapeutic efficacy by improving antigen presentation and tumor microenvironment modulation. Notably, responses were not significantly different across histologic subtypes, supporting the use of PC across squamous and non-squamous subtypes of NSCLC. In patients with squamous NSCLC, the PC combination led to ORR and PFS improvements comparable to those observed in non-squamous patients. Furthermore, real-world studies from Japan and South Korea have shown that Asian patients may have heightened immunogenic responses to ICIs than their Western counterparts do, potentially due to differences in tumor mutational burden (TMB), the gut microbiome, and HLA profiles (56, 57).

The enhanced efficacy of PC therapy is associated with a higher incidence of AEs, particularly irAEs. Furthermore, TEAEs leading to discontinuation were more common in the PC group (RR: 1.66). This safety profile is consistent with prior studies. In the IMpower132 trial, grade ≥3 TRAEs occurred in 47.2% of individuals receiving atezolizumab versus 36.9% receiving chemotherapy (12). Similarly, in the ORIENT-12 trial, the rate of grade ≥3 TRAEs was 61.3% in the PC group (16). Importantly, while the overall incidence of AEs was elevated, treatment-related mortality did not differ significantly between groups, suggesting that these toxicities are manageable with timely intervention. Our meta-analysis revealed a significantly increased risk of grade 3–5 irAEs, particularly pneumonitis (RR: 3.12, 1.81% vs. 0.64%), rash (RR: 2.13, 1.08% vs. 0.78%), hypothyroidism (RR: 4.69, 1.63% vs. 0.46%), and hepatitis (RR: 1.92, 1.21% vs. 0.46%), in the PC group. Pneumonitis is one of the most clinically concerning toxicities because of its potential severity and overlap with infection or radiation pneumonitis (58). Although colitis was not frequently reported across the included trials, its occurrence in real-world settings has been recognized as a critical irAE. Prompt recognition and early intervention, typically involving corticosteroids and temporary or permanent discontinuation of immunotherapy, are essential to mitigate these risks. Furthermore, we emphasize the need for multidisciplinary collaboration, including pulmonology, endocrinology, and gastroenterology support, to manage such irAEs effectively (59). Importantly, treatment-related mortality did not differ significantly between groups, indicating that, while irAEs are more frequent with PC, they are manageable with appropriate surveillance and intervention. Interestingly, some studies suggest a paradoxical relationship between irAEs and improved survival outcomes. Patients who develop irAEs tend to have more durable responses, possibly reflecting stronger immune activation (60). In Asian cohorts, this phenomenon appears even more pronounced. In a retrospective study in China, NSCLC patients who developed irAEs had significantly longer OS and PFS (61). However, the increased incidence of irAEs also underscores the need for multidisciplinary management and early immunotherapy-specific toxicity education in clinical settings. Moreover, it remains unclear whether certain genetic or pharmacogenomic factors predispose Asian patients to higher irAE rates. Future pharmacovigilance studies in real-world Asian populations could provide valuable insights into risk stratification and prevention strategies for irAEs.

Our pooled results have several direct implications for clinical practice and health policy decision-making in Asian settings. First, although PC therapy provides clear survival and response benefits, the high acquisition cost of ICIs and variability in national reimbursement policies across Asian countries may limit equitable access (62). Second, cost-effectiveness is likely to vary by country, tumor histology, PD-L1 expression, and competing domestic pricing/reimbursement frameworks; therefore, region-specific economic evaluations are urgently needed to inform reimbursement and guideline decisions (63). Third, differential access (urban vs. rural centers, tertiary vs. community hospitals) and local drug availability should be considered when translating trial results into practice; implementation strategies (including biomarker-guided selection and optimized treatment sequencing) may help maximize benefit within constrained resources (64). Finally, real-world pharmacovigilance and prospective health-economic studies in diverse Asian populations are recommended to quantify the value, affordability, and scalability of first-line PC regimens and to inform policy and clinical guideline development (65).

Future studies should focus on real-world pharmacovigilance to better characterize irAEs and treatment tolerability among diverse Asian subpopulations. Additionally, region-specific cost-effectiveness analyses are warranted to inform equitable access and reimbursement strategies, as the high cost and variable availability of ICIs remain significant challenges in many Asian healthcare systems. Multi-omics and translational studies investigating ethnic differences in immune gene expression, tumor mutational burden, and host microbiome composition may further clarify the biological mechanisms underlying the differential efficacy observed in Asian patients.

Despite these robust findings, our meta-analysis has certain limitations. First, the inclusion of studies with varying designs, patient populations, and treatment regimens may introduce heterogeneity. Second, the definition of the Asian population is broad. Some trials enrolled only Chinese (East Asian) patients, while others provided Asian subgroup data without specifying regions. Thus, our analysis cannot differentiate between East, South, or West Asian populations, and future region-specific studies are needed to clarify intra-Asian heterogeneity in immunotherapy outcomes. Third, the number of patients with brain metastases in the included studies was limited, which may reduce the statistical robustness and generalizability of the observed survival benefits in this subgroup. Additionally, the retrospective nature of subgroup analyses limits the ability to draw definitive conclusions for specific patient subsets. The potential for publication bias exists, as studies with negative results may be underreported. Furthermore, the assessment of adverse events relied on reported data from the included trials, which may not capture the full spectrum of real-world toxicities. Finally, the meta-analysis did not evaluate the cost-effectiveness of PC, an essential factor for global healthcare decision-making.

## Conclusion

PD-1/PD-L1 inhibitors plus chemotherapy enhance survival benefits in Asian patients with stage IIIb–IV NSCLC. The PC regimen improves response rates and offers durable tumor control. However, the increase in AEs in the PC group, particularly irAEs, highlights the importance of close surveillance and proper management. These findings support the integration of ICIs into first-line treatment regimens for advanced NSCLC in Asian populations, with careful consideration of individual patient factors and potential toxicities. However, country-level cost, reimbursement, and access considerations may affect implementation in Asian health systems; region-specific economic and real-world studies are warranted to guide equitable adoption.

## Data Availability

The original contributions presented in the study are included in the article/**Supplementary Material**. Further inquiries can be directed to the corresponding author.

## Supplementary material

Supplementary Figure 1 Cochrane Risk Assessment.

Supplementary Figure 2 Forest plots of OSR at 6–60 months associated with PC versus chemotherapy.

Supplementary Figure 3 Forest plots of PFSR at 6–60 months associated with PC versus chemotherapy.

Supplementary Figure 4 Forest plots of responses associated with PC versus chemotherapy.

Supplementary Figure 5 Forest plots of DORR at 6–48 months associated with PC versus chemotherapy.

Supplementary Figure 6 Sensitivity analysis of PFSR-6m **(A)**, stable disease **(B)**, and grade 3–5 TEAEs **(C)**.

Supplementary Figure 7 Funnel plots of OS **(A)**, PFS **(B)**, ORR **(C)**, and total TEAEs **(D)**.

Supplementary Figure 8 Egger’s and Begg’s tests of OS **(A)**, PFS **(B)**, ORR **(C)**, and grade TEAEs **(D)**.

## Glossary

AE: adverse event
ALT: alanine aminotransferase
AST: aspartate aminotransferase
CPS: combined positive score
CR: complete response
CI: confidence interval
DCR: disease control rate
DOR: duration of response
DORR: duration of response rate
EGFR: epidermal growth factor receptor
ECOG PS: Eastern Cooperative Oncology Group Performance Status
GRADE: Grading of Recommendations, Assessment, Development, and Evaluation
HLA: human leukocyte antigen
HR: hazard ratio
ICIs: immune checkpoint inhibitors
irAE: immune-related adverse event
M/F: male/female
NSCLC: non-small-cell lung cancer
ORR: objective response rate
OS: overall survival
OSR: overall survival rate
*p*: probability
PC: PD-1/PD-L1 inhibitors combined with chemotherapy
PD: progressive disease
PD-1: programmed death-1
PD-L1: programmed death-ligand 1
PFS: progression-free survival
PFSR: progression-free survival rate
PR: partial response
PRISMA: Preferred Reporting Items for Systematic Reviews and Meta-Analyses
RCT: randomized controlled trial
RR: risk ratio
SD: stable disease
TEAE: treatment-emergent adverse event
TRAEs: treatment-related adverse events
TMB: tumor mutational burden
