# Supplementary figures and images for: First-line immunochemotherapy for advanced NSCLC in Asian patients: a meta-analysis of phase 3 RCTs
Source: Front Oncol. 2025 Nov 19;15:1709348. doi: 10.3389/fonc.2025.1709348 (PMC12672283; doi:10.3389/fonc.2025.1709348)

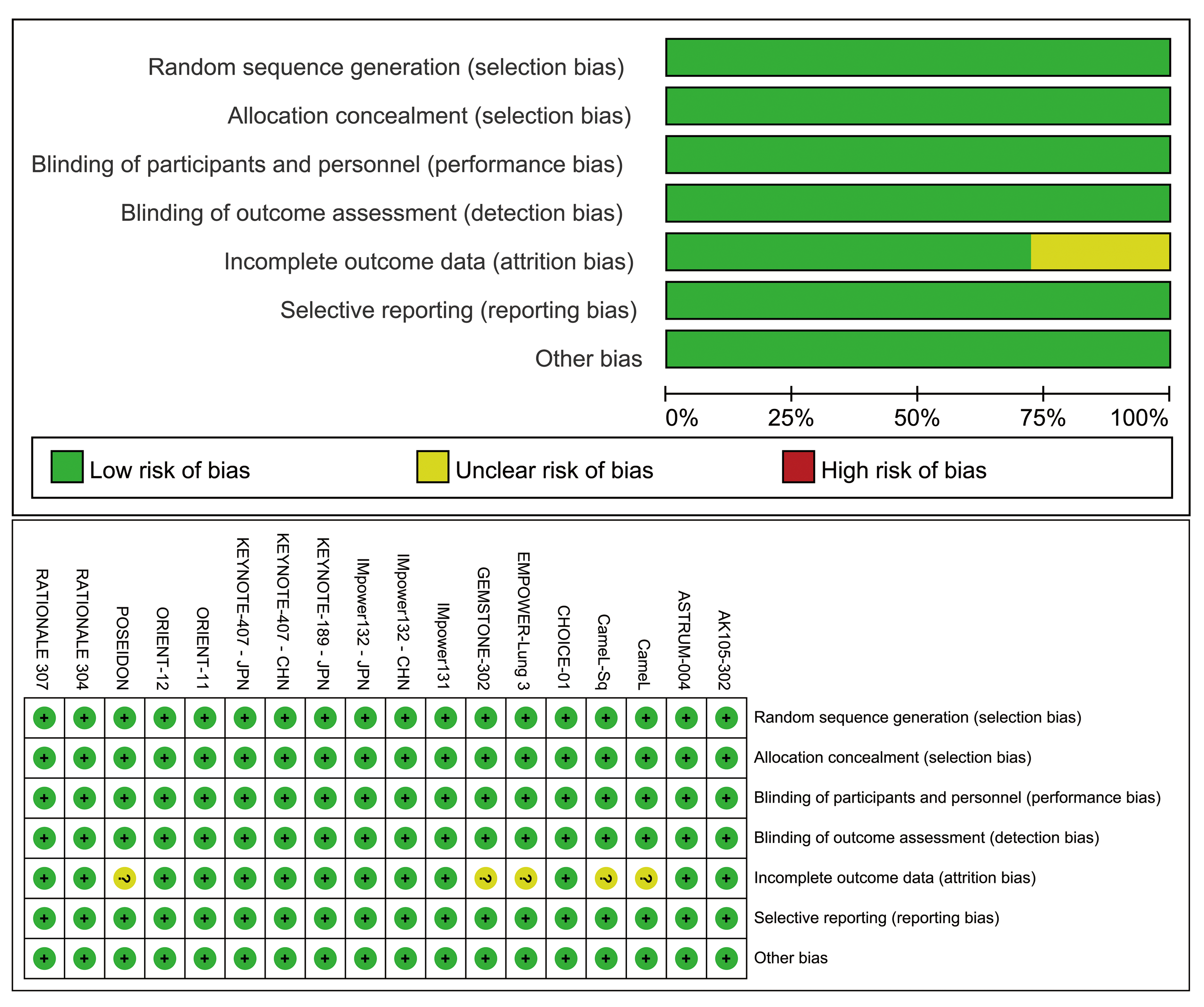

Supplement: Supplementary Figure 1 — Cochrane Risk Assessment. [file Image1.tif]

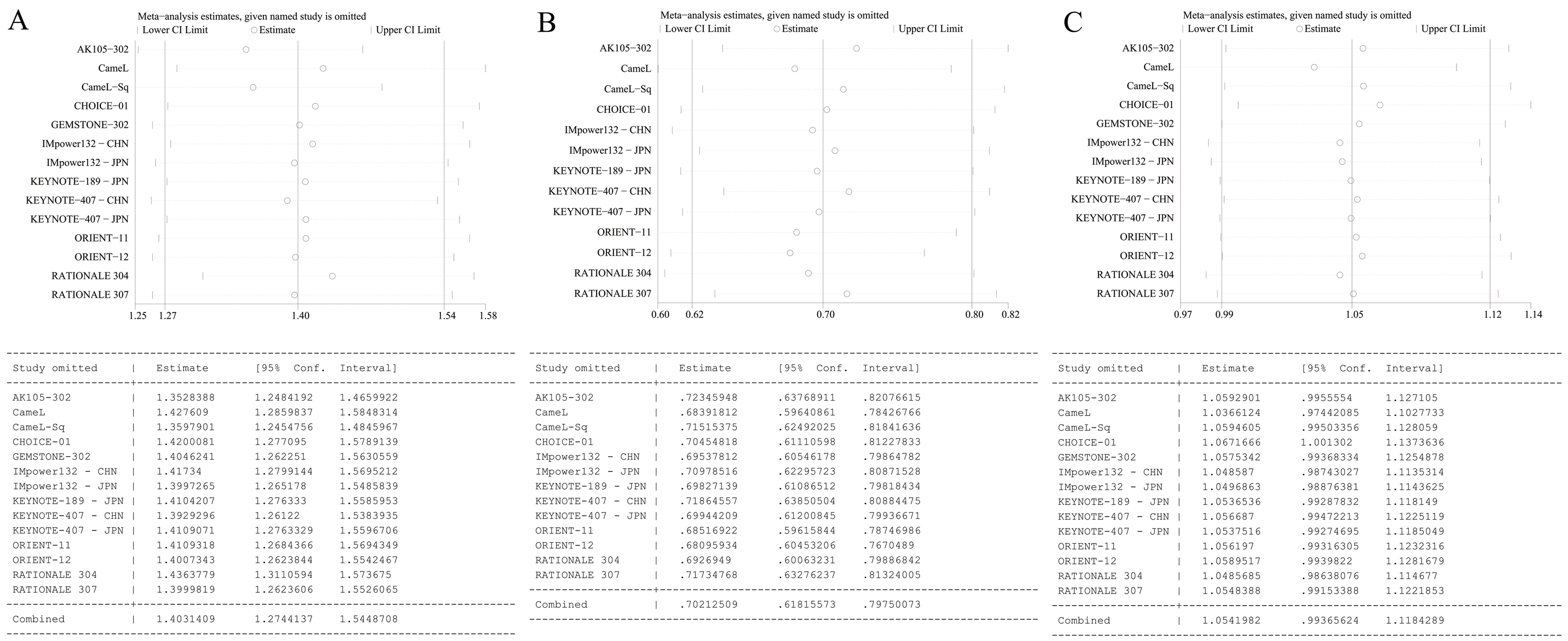

Supplement: Supplementary Figure 6 — Sensitivity analysis of PFSR-6m (A), stable disease (B), and grade 3–5 TEAEs (C). [file Image6.tif]

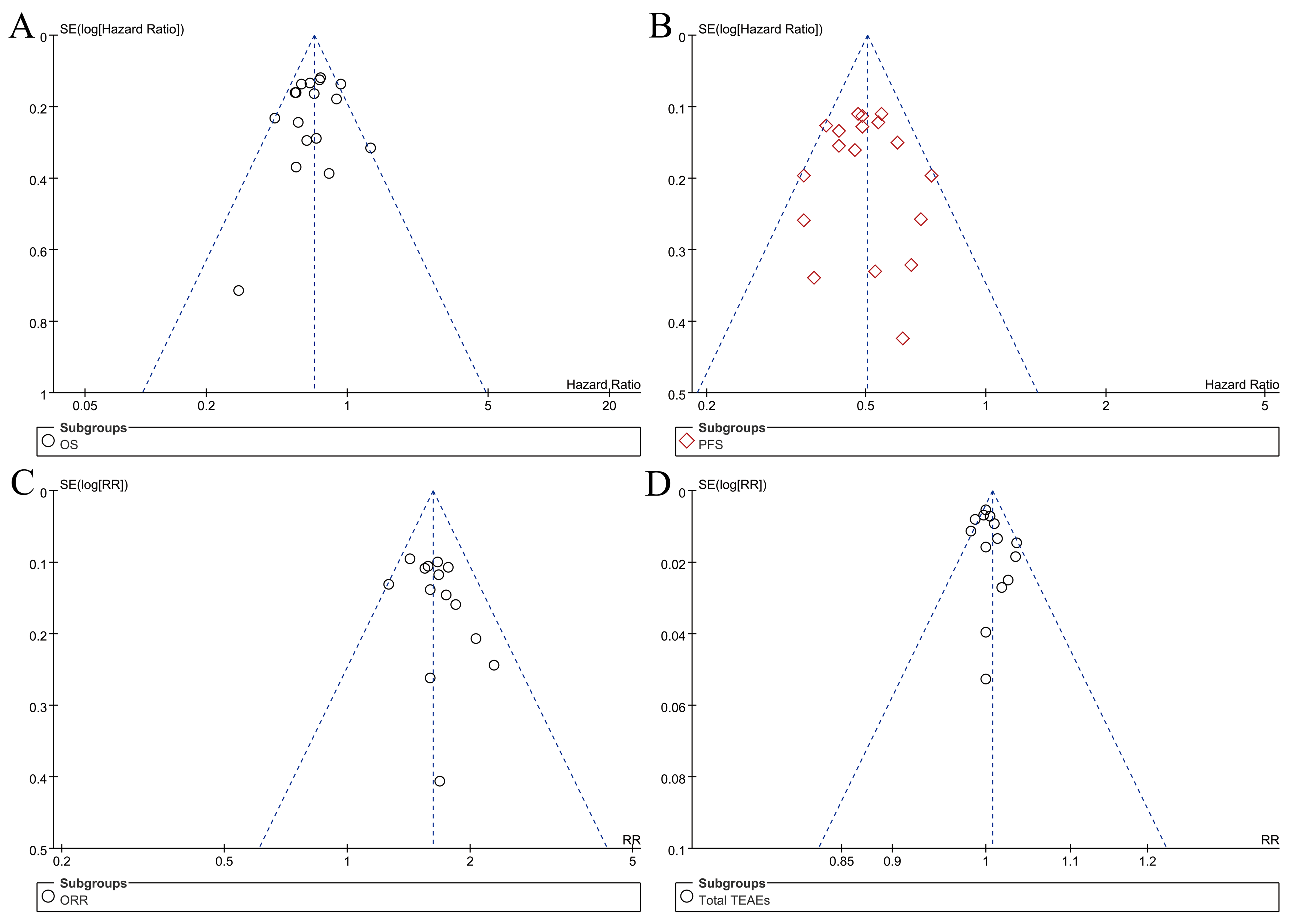

Supplement: Supplementary Figure 7 — Funnel plots of OS (A), PFS (B), ORR (C), and total TEAEs (D). [file Image7.tif]

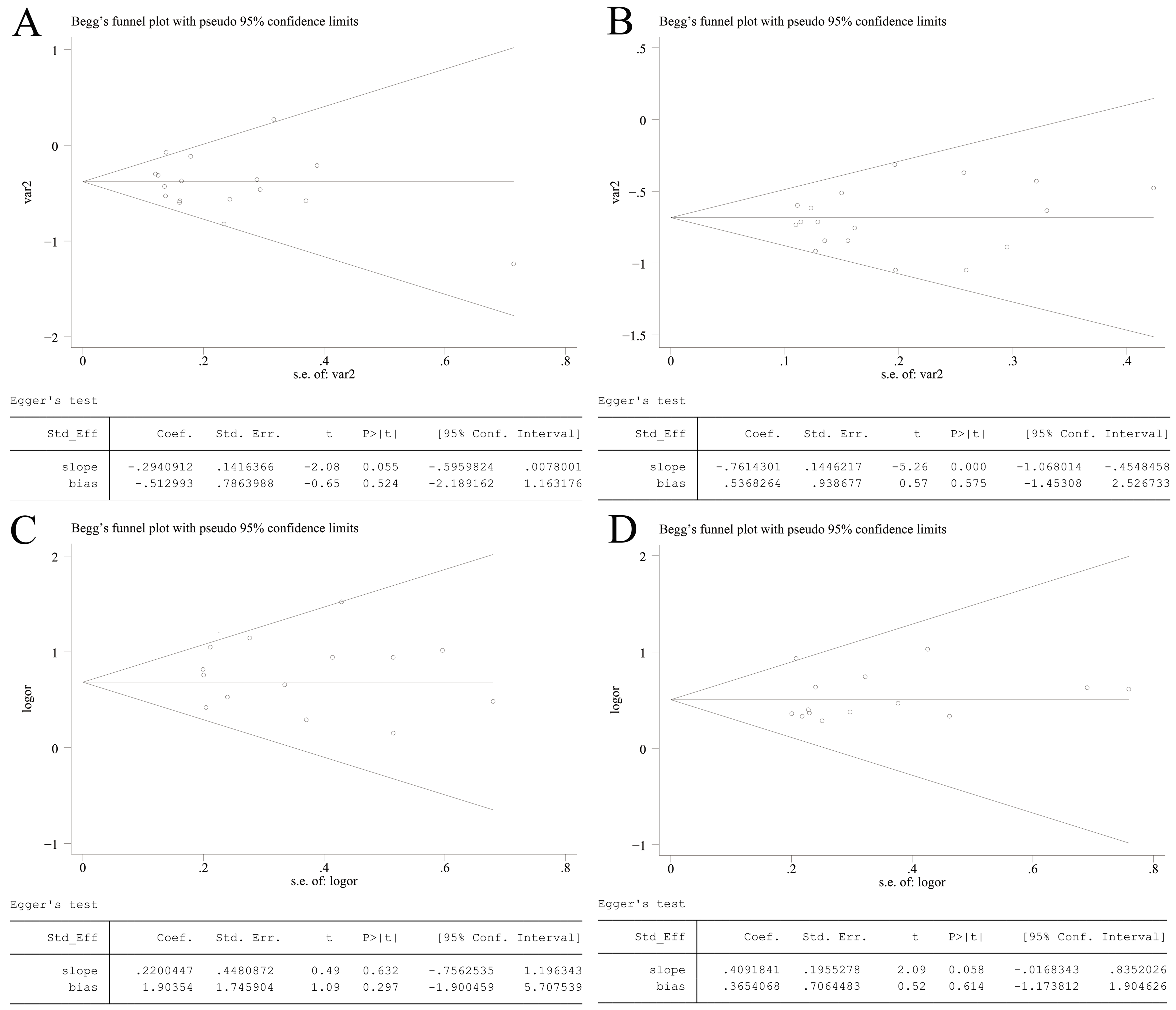

Supplement: Supplementary Figure 8 — Egger’s and Begg’s tests of OS (A), PFS (B), ORR (C), and grade TEAEs (D). [file Image8.tif]
