# Supplementary material for: First-line immunochemotherapy for advanced NSCLC in Asian patients: a meta-analysis of phase 3 RCTs
Source: Front Oncol. 2025 Nov 19;15:1709348. doi: 10.3389/fonc.2025.1709348 (PMC12672283; doi:10.3389/fonc.2025.1709348)
Supplement: Supplementary file 10 [file Table2.doc]

**Table S2 Methodological quality assessments (Jadad scale) of the included studies.**

| **Study** | **Registration No.** | **Randomization** | **Concealment of allocation** | **Double blinding** | **Withdrawals and dropouts** | **Quality (score)** |
| --- | --- | --- | --- | --- | --- | --- |
| AK105-302 (4) | NCT03866993 | ** | ** | * | * | 7 |
| ASTRUM-004 (5) | NCT04033354 | ** | ** | * | * | 7 |
| CameL (6,31,32) | NCT03134872 | ** | ** | * | * | 7 |
| CameL-Sq (7) | NCT03668496 | ** | ** | * | * | 7 |
| CHOICE-01 (8,33) | NCT03856411 | ** | ** | * | * | 7 |
| EMPOWER-Lung 3 (9,34,35) | NCT03409614 | ** | ** | * | * | 7 |
| GEMSTONE-302 (10,36) | NCT03789604 | ** | ** | * | * | 7 |
| IMpower131 (11) | NCT02367794 | ** | ** | * | * | 7 |
| IMpower132 (12,37,38) | NCT02657434 | ** | ** | * | * | 7 |
| KEYNOTE-189 (13,39-43) | NCT02578680 | ** | ** | * | * | 7 |
| KEYNOTE-407 (14,44-48) | NCT02775435 | ** | ** | * | * | 7 |
| ORIENT-11 (15,49,50) | NCT03607539 | ** | ** | * | * | 7 |
| ORIENT-12 (16) | NCT03629925 | ** | ** | * | * | 7 |
| POSEIDON (17,51) | NCT03164616 | ** | ** | * | * | 7 |
| RATIONALE 304 (18,52) | NCT03663205 | ** | ** | * | * | 7 |
| RATIONALE 307 (19,53) | NCT03594747 | ** | ** | * | * | 7 |
