# Supplementary material for: First-line immunochemotherapy for advanced NSCLC in Asian patients: a meta-analysis of phase 3 RCTs
Source: Front Oncol. 2025 Nov 19;15:1709348. doi: 10.3389/fonc.2025.1709348 (PMC12672283; doi:10.3389/fonc.2025.1709348)
Supplement: Supplementary file 11 [file Table3.doc]

**Table S3** GRADE quality assessment by therapeutic strategy and study design for the outcomes.

| **Outcomes** | **No. of Participants** | | **Differences (95%CI) a** | **Quality Assessment** | | | | | **Quality** |
| --- | --- | --- | --- | --- | --- | --- | --- | --- | --- |
|  | **PC** | **Chemotherapy** |  | **Risk of Biasb** | **Inconsistency** | **Indirectness** | **Imprecision** | **Publication Biasc** |  |
| **Survival** |  |  |  |  |  |  |  |  |  |
| OS | 4293 |  | 0.68 [0.63, 0.75] | Low | No inconsistency | No indirectness | No imprecision | Unlikely | High |
| PFS | 4452 |  | 0.50 [0.47, 0.54] | Low | No inconsistency | No indirectness | No imprecision | Unlikely | High |
| **Survival rate** |  |  |  |  |  |  |  |  |  |
| **OSR** |  |  |  |  |  |  |  |  |  |
| OSR-6m | 2008/2232 | 1411/1671 | 1.07 [1.04, 1.10] | Low | No inconsistency | No indirectness | No imprecision | Unlikely | High |
| OSR-12m | 1665/2232 | 1090/1671 | 1.15 [1.10, 1.20] | Low | No inconsistency | No indirectness | No imprecision | Unlikely | High |
| OSR-18m | 1268/2053 | 730/1493 | 1.27 [1.19, 1.35] | Low | No inconsistency | No indirectness | No imprecision | Unlikely | High |
| OSR-24m | 859/1628 | 431/1180 | 1.46 [1.34, 1.59] | Low | No inconsistency | No indirectness | No imprecision | Unlikely | High |
| OSR-30m | 363/802 | 191/651 | 1.63 [1.28, 2.08] | Low | Serious (-1) | No indirectness | No imprecision | Unlikely | Medium |
| OSR-36m | 179/514 | 90/363 | 1.48 [1.19, 1.85] | Low | No inconsistency | No indirectness | No imprecision | Unlikely | High |
| OSR-42m | 78/205 | 52/207 | 1.51 [1.13, 2.03] | Low | No inconsistency | No indirectness | No imprecision | Unlikely | High |
| OSR-48m | 73/205 | 52/207 | 1.42 [1.05, 1.91] | Low | No inconsistency | No indirectness | No imprecision | Unlikely | High |
| OSR-54m | 67/205 | 45/207 | 1.50 [1.09, 2.08] | Low | No inconsistency | No indirectness | No imprecision | Unlikely | High |
| OSR-60m | 61/205 | 37/207 | 1.66 [1.16, 2.39] | Low | No inconsistency | No indirectness | No imprecision | Unlikely | High |
| **PFSR** |  |  |  |  |  |  |  |  |  |
| PFSR-6m | 1435/2232 | 632/1671 | 1.66 [1.46, 1.88] | Low | Serious (-1) | No indirectness | No imprecision | Unlikely | Medium |
| PFSR-12m | 823/2232 | 231/1671 | 2.67 [2.11, 3.39] | Low | No inconsistency | No indirectness | No imprecision | Unlikely | High |
| PFSR-18m | 550/1971 | 112/1412 | 3.53 [2.91, 4.28] | Low | No inconsistency | No indirectness | No imprecision | Unlikely | High |
| PFSR-24m | 181/754 | 35/598 | 4.69 [1.94, 11.30] | Low | Serious (-1) | No indirectness | No imprecision | Unlikely | Medium |
| PFSR-30m | 48/205 | 13/207 | 3.73 [2.08, 6.67] | Low | No inconsistency | No indirectness | No imprecision | Unlikely | High |
| PFSR-36m | 40/205 | 10/207 | 4.04 [2.08, 7.86] | Low | No inconsistency | No indirectness | No imprecision | Unlikely | High |
| PFSR-42m | 34/205 | 8/207 | 4.29 [2.04, 9.04] | Low | No inconsistency | No indirectness | No imprecision | Unlikely | High |
| PFSR-48m | 33/205 | 5/207 | 6.66 [2.65, 16.73] | Low | No inconsistency | No indirectness | No imprecision | Unlikely | High |
| PFSR-54m | 33/205 | 5/207 | 6.66 [2.65, 16.73] | Low | No inconsistency | No indirectness | No imprecision | Unlikely | High |
| PFSR-60m | 33/205 | 5/207 | 6.66 [2.65, 16.73] | Low | No inconsistency | No indirectness | No imprecision | Unlikely | High |
| **Subgroup analysis of OS** |  |  |  |  |  |  |  |  |  |
| Total | 4293 | | 0.68 [0.63, 0.75] | Low | No inconsistency | No indirectness | No imprecision | Unlikely | High |
| Age - < 65 years | 1479 | | 0.64 [0.56, 0.74] | Low | No inconsistency | No indirectness | No imprecision | Unlikely | High |
| Age - > 65 years | 868 | | 0.78 [0.65, 0.93] | Low | No inconsistency | No indirectness | No imprecision | Unlikely | High |
| Sex - Female | 416 | | 0.75 [0.56, 1.00] | Low | No inconsistency | No indirectness | No imprecision | Unlikely | High |
| Sex - Male | 1581 | | 0.69 [0.61, 0.79] | Low | No inconsistency | No indirectness | No imprecision | Unlikely | High |
| ECOG PS - 0 | 542 | | 0.62 [0.48, 0.80] | Low | No inconsistency | No indirectness | No imprecision | Unlikely | High |
| ECOG PS - 1 | 1805 | | 0.71 [0.62, 0.80] | Low | No inconsistency | No indirectness | No imprecision | Unlikely | High |
| Smoking status - Current/former | 1443 | | 0.60 [0.52, 0.70] | Low | No inconsistency | No indirectness | No imprecision | Unlikely | High |
| Smoking status - Never | 492 | | 0.88 [0.67, 1.16] | Low | No inconsistency | No indirectness | No imprecision | Unlikely | High |
| Pathological type - Squamous | 2042 | | 0.64 [0.52, 0.80] | Low | No inconsistency | No indirectness | No imprecision | Unlikely | High |
| Pathological type - Non-squamous | 1979 | | 0.68 [0.60, 0.77] | Low | No inconsistency | No indirectness | No imprecision | Unlikely | High |
| Stage - Stage III | 327 | | 0.78 [0.55, 1.11] | Low | No inconsistency | No indirectness | No imprecision | Unlikely | High |
| Stage - Stage IV | 2895 | | 0.66 [0.59, 0.73] | Low | No inconsistency | No indirectness | No imprecision | Unlikely | High |
| Brain metastases - Yes | 75 | | 0.54 [0.29, 1.03] | Low | No inconsistency | No indirectness | No imprecision | Unlikely | High |
| Brain metastases - No | 734 | | 0.68 [0.57, 0.81] | Low | No inconsistency | No indirectness | No imprecision | Unlikely | High |
| PD-L1 CPS - <1% | 817 | | 0.80 [0.67, 0.96] | Low | No inconsistency | No indirectness | No imprecision | Unlikely | High |
| PD-L1 CPS - >1% | 1134 | | 0.59 [0.50, 0.70] | Low | No inconsistency | No indirectness | No imprecision | Unlikely | High |
| PD-L1 CPS - 1%-49% | 764 | | 0.70 [0.57, 0.85] | Low | No inconsistency | No indirectness | No imprecision | Unlikely | High |
| PD-L1 CPS - >50% | 405 | | 0.54 [0.39, 0.74] | Low | No inconsistency | No indirectness | No imprecision | Unlikely | High |
| PD-1/PD-L1 inhibitors type - Penpulimab | 350 | | 0.55 [0.40, 0.75] | Low | No inconsistency | No indirectness | No imprecision | Unlikely | High |
| PD-1/PD-L1 inhibitors type - Sugemalimab | 479 | | 0.59 [0.45, 0.77] | Low | No inconsistency | No indirectness | No imprecision | Unlikely | High |
| PD-1/PD-L1 inhibitors type - Camrelizumab | 801 | | 0.67 [0.55, 0.81] | Low | No inconsistency | No indirectness | No imprecision | Unlikely | High |
| PD-1/PD-L1 inhibitors type - Toripalimab | 465 | | 0.73 [0.57, 0.93] | Low | No inconsistency | No indirectness | No imprecision | Unlikely | High |
| PD-1/PD-L1 inhibitors type - Cemiplimab | 61 | | 0.81 [0.38, 1.73] | Low | No inconsistency | No indirectness | No imprecision | Unlikely | High |
| PD-1/PD-L1 inhibitors type - Atezolizumab | 342 | | 0.81 [0.58, 1.14] | Low | No inconsistency | No indirectness | No imprecision | Unlikely | High |
| PD-1/PD-L1 inhibitors type - Pembrolizumab | 215 | | 0.46 [0.31, 0.66] | Low | No inconsistency | No indirectness | No imprecision | Unlikely | High |
| PD-1/PD-L1 inhibitors type - Sintilimab | 794 | | 0.63 [0.50, 0.79] | Low | No inconsistency | No indirectness | No imprecision | Unlikely | High |
| PD-1/PD-L1 inhibitors type - Durvalumab | 251 | | 0.93 [0.71, 1.22] | Low | No inconsistency | No indirectness | No imprecision | Unlikely | High |
| PD-1/PD-L1 inhibitors type - Tislelizumab | 575 | | 0.77 [0.61, 0.98] | Low | No inconsistency | No indirectness | No imprecision | Unlikely | High |
| Platinum chemotherapy type - Cisplatin | 104 | | 0.53 [0.32, 0.87] | Low | No inconsistency | No indirectness | No imprecision | Unlikely | High |
| Platinum chemotherapy type - Carboplatin | 2941 | | 0.67 [0.60, 0.74] | Low | No inconsistency | No indirectness | No imprecision | Unlikely | High |
| **Subgroup analysis of PFS** |  |  |  |  |  |  |  |  |  |
| Total | 4652 | | 0.50 [0.47, 0.54] | Low | No inconsistency | No indirectness | No imprecision | Unlikely | High |
| Age - < 65 years | 2082 | | 0.46 [0.41, 0.51] | Low | No inconsistency | No indirectness | No imprecision | Unlikely | High |
| Age - > 65 years | 1342 | | 0.55 [0.48, 0.62] | Low | No inconsistency | No indirectness | No imprecision | Unlikely | High |
| Sex - Female | 565 | | 0.65 [0.53, 0.80] | Low | No inconsistency | No indirectness | No imprecision | Unlikely | High |
| Sex - Male | 2509 | | 0.48 [0.43, 0.52] | Low | No inconsistency | No indirectness | No imprecision | Unlikely | High |
| ECOG PS - 0 | 739 | | 0.51 [0.42, 0.61] | Low | No inconsistency | No indirectness | No imprecision | Unlikely | High |
| ECOG PS - 1 | 2685 | | 0.49 [0.45, 0.54] | Low | No inconsistency | No indirectness | No imprecision | Unlikely | High |
| Smoking status - Current/former | 2290 | | 0.45 [0.40, 0.50] | Low | No inconsistency | No indirectness | No imprecision | Unlikely | High |
| Smoking status - Never | 722 | | 0.59 [0.49, 0.70] | Low | No inconsistency | No indirectness | No imprecision | Unlikely | High |
| Pathological type - Squamous | 2361 | | 0.45 [0.41, 0.50] | Low | No inconsistency | No indirectness | No imprecision | Unlikely | High |
| Pathological type - Non-squamous | 1979 | | 0.54 [0.49, 0.61] | Low | No inconsistency | No indirectness | No imprecision | Unlikely | High |
| Stage - Stage III | 492 | | 0.43 [0.34, 0.54] | Low | No inconsistency | No indirectness | No imprecision | Unlikely | High |
| Stage - Stage IV | 3328 | | 0.51 [0.47, 0.55] | Low | No inconsistency | No indirectness | No imprecision | Unlikely | High |
| Brain metastases - Yes | 142 | | 0.38 [0.24, 0.58] | Low | No inconsistency | No indirectness | No imprecision | Unlikely | High |
| Brain metastases - No | 1144 | | 0.53 [0.46, 0.61] | Low | No inconsistency | No indirectness | No imprecision | Unlikely | High |
| PD-L1 CPS - <1% | 1231 | | 0.61 [0.53, 0.70] | Low | No inconsistency | No indirectness | No imprecision | Unlikely | High |
| PD-L1 CPS - >1% | 1804 | | 0.44 [0.40, 0.50] | Low | No inconsistency | No indirectness | No imprecision | Unlikely | High |
| PD-L1 CPS - 1%-49% | 1079 | | 0.54 [0.47, 0.63] | Low | No inconsistency | No indirectness | No imprecision | Unlikely | High |
| PD-L1 CPS - >50% | 760 | | 0.40 [0.33, 0.49] | Low | No inconsistency | No indirectness | No imprecision | Unlikely | High |
| PD-1/PD-L1 inhibitors type - Penpulimab | 350 | | 0.43 [0.33, 0.56] | Low | No inconsistency | No indirectness | No imprecision | Unlikely | High |
| PD-1/PD-L1 inhibitors type - Sugemalimab | 838 | | 0.46 [0.39, 0.55] | Low | No inconsistency | No indirectness | No imprecision | Unlikely | High |
| PD-1/PD-L1 inhibitors type - Camrelizumab | 801 | | 0.47 [0.35, 0.64] | Low | Serious (-1) | No indirectness | No imprecision | Unlikely | Medium |
| PD-1/PD-L1 inhibitors type - Toripalimab | 465 | | 0.49 [0.39, 0.61] | Low | No inconsistency | No indirectness | No imprecision | Unlikely | High |
| PD-1/PD-L1 inhibitors type - Cemiplimab | 61 | | 0.53 [0.28, 1.01] | Low | No inconsistency | No indirectness | No imprecision | Unlikely | High |
| PD-1/PD-L1 inhibitors type - Atezolizumab | 342 | | 0.57 [0.36, 0.89] | Low | Serious (-1) | No indirectness | No imprecision | Unlikely | Medium |
| PD-1/PD-L1 inhibitors type - Pembrolizumab | 215 | | 0.44 [0.32, 0.60] | Low | No inconsistency | No indirectness | No imprecision | Unlikely | High |
| PD-1/PD-L1 inhibitors type - Sintilimab | 754 | | 0.52 [0.43, 0.61] | Low | No inconsistency | No indirectness | No imprecision | Unlikely | High |
| PD-1/PD-L1 inhibitors type - Durvalumab | 251 | | 0.77 [0.57, 1.04] | Low | No inconsistency | No indirectness | No imprecision | Unlikely | High |
| PD-1/PD-L1 inhibitors type - Tislelizumab | 572 | | 0.54 [0.43, 0.66] | Low | No inconsistency | No indirectness | No imprecision | Unlikely | High |
| Platinum chemotherapy type - Cisplatin | 239 | | 0.55 [0.41, 0.73] | Low | No inconsistency | No indirectness | No imprecision | Unlikely | High |
| Platinum chemotherapy type - Carboplatin | 3522 | | 0.48 [0.44, 0.52] | Low | No inconsistency | No indirectness | No imprecision | Unlikely | High |
| **Responses** |  |  |  |  |  |  |  |  |  |
| ORR | 1362/2232 | 631/1671 | 1.62 [1.51, 1.74] | Low | No inconsistency | No indirectness | No imprecision | Unlikely | High |
| DCR | 1694/1912 | 1231/1512 | 1.09 [1.05, 1.12] | Low | No inconsistency | No indirectness | No imprecision | Unlikely | High |
| CR | 45/1912 | 11/1512 | 3.21 [1.78, 5.78] | Low | No inconsistency | No indirectness | No imprecision | Unlikely | High |
| PR | 1114/1912 | 558/1512 | 1.58 [1.46, 1.70] | Low | No inconsistency | No indirectness | No imprecision | Unlikely | High |
| SD | 536/1912 | 662/1512 | 0.61 [0.52, 0.72] | Low | Serious (-1) | No indirectness | No imprecision | Unlikely | Medium |
| PD | 136/1912 | 199/1512 | 0.55 [0.45, 0.68] | Low | No inconsistency | No indirectness | No imprecision | Unlikely | High |
| DOR | 1202 | 893 | 0.43 [0.36, 0.50] | Low | No inconsistency | No indirectness | No imprecision | Unlikely | High |
| **DORR** |  |  |  |  |  |  |  |  |  |
| DORR-6m | 313/441 | 85/204 | 1.78 [1.11, 2.86] | Low | Serious (-1) | No indirectness | No imprecision | Unlikely | Medium |
| DORR-12m | 215/441 | 50/204 | 2.14 [1.22, 3.78] | Low | Serious (-1) | No indirectness | No imprecision | Unlikely | Medium |
| DORR-18m | 118/316 | 22/132 | 2.56 [1.12, 5.84] | Low | Serious (-1) | No indirectness | No imprecision | Unlikely | Medium |
| DORR-24m | 97/316 | 14/132 | 3.13 [1.85, 5.29] | Low | No inconsistency | No indirectness | No imprecision | Unlikely | High |
| DORR-30m | 40/113 | 8/68 | 3.01 [1.50, 6.04] | Low | No inconsistency | No indirectness | No imprecision | Unlikely | High |
| DORR-36m | 30/113 | 8/68 | 2.26 [1.10, 4.63] | Low | No inconsistency | No indirectness | No imprecision | Unlikely | High |
| DORR-42m | 27/113 | 5/68 | 3.25 [1.31, 8.04] | Low | No inconsistency | No indirectness | No imprecision | Unlikely | High |
| DORR-48m | 27/113 | 5/68 | 3.25 [1.31, 8.04] | Low | No inconsistency | No indirectness | No imprecision | Unlikely | High |
| **Safety summary** |  |  |  |  |  |  |  |  |  |
| Total TEAEs | 2220/2232 | 1650/1671 | 1.01 [1.00, 1.01] | Low | No inconsistency | No indirectness | No imprecision | Unlikely | High |
| Grade 3-5 TEAEs | 1615/2232 | 1117/1671 | 1.10 [1.03, 1.18] | Low | Serious (-1) | No indirectness | No imprecision | Unlikely | Medium |
| Serious TEAEs | 639/1675 | 316/1259 | 1.54 [1.24, 1.91] | Low | Serious (-1) | No indirectness | No imprecision | Unlikely | Medium |
| TEAEs leading to discontinuation | 343/2232 | 154/1671 | 1.66 [1.21, 2.27] | Low | Serious (-1) | No indirectness | No imprecision | Unlikely | Medium |
| TEAEs leading to death | 129/2232 | 104/1671 | 1.01 [0.78, 1.30] | Low | No inconsistency | No indirectness | No imprecision | Unlikely | High |
| Total TRAEs | 1219/1230 | 1053/1080 | 1.01 [1.00, 1.03] | Low | Serious (-1) | No indirectness | No imprecision | Unlikely | Medium |
| Grade 3-5 TRAEs | 825/1208 | 655/1052 | 1.13 [1.01, 1.26] | Low | Serious (-1) | No indirectness | No imprecision | Unlikely | Medium |
| Serious TRAEs | 305/1023 | 172/871 | 1.57 [1.14, 2.17] | Low | Serious (-1) | No indirectness | No imprecision | Unlikely | Medium |
| TRAEs leading to discontinuation | 96/958 | 31/797 | 2.26 [1.53, 3.32] | Low | No inconsistency | No indirectness | No imprecision | Unlikely | High |
| TRAEs leading to death | 37/1110 | 23/959 | 1.44 [0.86, 2.44] | Low | No inconsistency | No indirectness | No imprecision | Unlikely | High |
| Total irAEs | 760/1812 | 248/1240 | 2.53 [1.72, 3.73] | Low | Serious (-1) | No indirectness | No imprecision | Unlikely | Medium |
| Grade 3-5 irAEs | 148/1764 | 32/1187 | 2.69 [1.41, 5.14] | Low | Serious (-1) | No indirectness | No imprecision | Unlikely | Medium |
| Serious irAEs | 11/175 | 2/175 | 5.50 [1.24, 24.45] | Low | No inconsistency | No indirectness | No imprecision | Unlikely | High |
| irAEs leading to discontinuation | 6/175 | 1/175 | 6.00 [0.73, 49.32] | Low | No inconsistency | No indirectness | No imprecision | Unlikely | High |
| **TEAEs** |  |  |  |  |  |  |  |  |  |
| **Any grade** |  |  |  |  |  |  |  |  |  |
| Anaemia | 1700/2232 | 1218/1671 | 1.02 [0.99, 1.06] | Low | No inconsistency | No indirectness | No imprecision | Unlikely | High |
| Neutrophil count decreased | 1166/1700 | 918/1404 | 1.06 [1.01, 1.12] | Low | No inconsistency | No indirectness | No imprecision | Unlikely | High |
| White blood cell decreased | 1138/1700 | 903/1404 | 1.06 [1.00, 1.11] | Low | No inconsistency | No indirectness | No imprecision | Unlikely | High |
| Alopecia | 463/920 | 411/754 | 1.05 [0.97, 1.14] | Low | No inconsistency | No indirectness | No imprecision | Unlikely | High |
| Leukopenia | 549/1101 | 299/671 | 1.04 [0.96, 1.13] | Low | No inconsistency | No indirectness | No imprecision | Unlikely | High |
| Neutropenia | 554/1149 | 314/724 | 1.05 [0.90, 1.22] | Low | Serious (-1) | No indirectness | No imprecision | Unlikely | Medium |
| Thrombocytopenia | 434/1019 | 234/590 | 1.02 [0.92, 1.13] | Low | No inconsistency | No indirectness | No imprecision | Unlikely | High |
| Platelet count decreased | 682/1675 | 530/1389 | 1.08 [0.99, 1.18] | Low | No inconsistency | No indirectness | No imprecision | Unlikely | High |
| Alanine aminotransferase increased | 821/2210 | 494/1643 | 1.19 [1.09, 1.31] | Low | No inconsistency | No indirectness | No imprecision | Unlikely | High |
| Aspartate aminotransferase increased | 781/2210 | 410/1643 | 1.38 [1.16, 1.63] | Low | Serious (-1) | No indirectness | No imprecision | Unlikely | Medium |
| Nausea | 776/2232 | 534/1671 | 1.08 [0.98, 1.18] | Low | No inconsistency | No indirectness | No imprecision | Unlikely | High |
| Hypoesthesia | 191/553 | 160/552 | 1.19 [1.00, 1.41] | Low | No inconsistency | No indirectness | No imprecision | Unlikely | High |
| Decreased appetite | 745/2232 | 496/1671 | 1.13 [1.02, 1.24] | Low | No inconsistency | No indirectness | No imprecision | Unlikely | High |
| Asthenia | 371/1365 | 272/1073 | 1.10 [0.96, 1.26] | Low | No inconsistency | No indirectness | No imprecision | Unlikely | High |
| Constipation | 499/2039 | 357/1475 | 1.05 [0.94, 1.18] | Low | No inconsistency | No indirectness | No imprecision | Unlikely | High |
| Fatigue | 284/1204 | 136/778 | 1.24 [0.86, 1.80] | Low | Serious (-1) | No indirectness | No imprecision | Unlikely | Medium |
| Pyrexia | 270/1244 | 154/959 | 1.36 [1.14, 1.63] | Low | No inconsistency | No indirectness | No imprecision | Unlikely | High |
| Vomiting | 459/2210 | 326/1643 | 1.03 [0.91, 1.17] | Low | No inconsistency | No indirectness | No imprecision | Unlikely | High |
| Hypoalbuminaemia | 300/1560 | 151/994 | 1.27 [1.06, 1.52] | Low | No inconsistency | No indirectness | No imprecision | Unlikely | High |
| Cough | 113/614 | 49/348 | 1.26 [0.92, 1.71] | Low | No inconsistency | No indirectness | No imprecision | Unlikely | High |
| Rash | 318/1743 | 118/1429 | 2.13 [1.75, 2.59] | Low | No inconsistency | No indirectness | No imprecision | Unlikely | High |
| Pain in extremity | 255/1430 | 180/993 | 1.07 [0.90, 1.27] | Low | No inconsistency | No indirectness | No imprecision | Unlikely | High |
| Arthralgia | 105/623 | 91/633 | 1.17 [0.91, 1.51] | Low | No inconsistency | No indirectness | No imprecision | Unlikely | High |
| Myalgia | 18/113 | 9/113 | 1.91 [0.91, 4.01] | Low | No inconsistency | No indirectness | No imprecision | Unlikely | High |
| Dysgeusia | 11/73 | 10/68 | 0.83 [0.18, 3.96] | Low | Serious (-1) | No indirectness | No imprecision | Unlikely | Medium |
| Malaise | 88/617 | 71/457 | 1.12 [0.85, 1.49] | Low | No inconsistency | No indirectness | No imprecision | Unlikely | High |
| Gamma-glutamyltransferase increased | 136/975 | 93/818 | 1.22 [0.83, 1.81] | Low | Serious (-1) | No indirectness | No imprecision | Unlikely | Medium |
| Weight decreased | 149/1077 | 112/795 | 1.01 [0.67, 1.54] | Low | Serious (-1) | No indirectness | No imprecision | Unlikely | Medium |
| Diarrhea | 154/1174 | 86/863 | 1.41 [1.10, 1.81] | Low | No inconsistency | No indirectness | No imprecision | Unlikely | High |
| Hypothyroidism | 133/1027 | 15/718 | 6.01 [3.56, 10.12] | Low | No inconsistency | No indirectness | No imprecision | Unlikely | High |
| Hyperglycemia | 89/694 | 34/375 | 1.47 [1.01, 2.12] | Low | No inconsistency | No indirectness | No imprecision | Unlikely | High |
| Hyponatraemia | 147/1185 | 103/870 | 1.18 [0.93, 1.50] | Low | No inconsistency | No indirectness | No imprecision | Unlikely | High |
| Hypokalaemia | 117/951 | 71/631 | 1.13 [0.86, 1.48] | Low | No inconsistency | No indirectness | No imprecision | Unlikely | High |
| Hyperthyroidism | 47/391 | 4/237 | 6.96 [2.55, 18.98] | Low | No inconsistency | No indirectness | No imprecision | Unlikely | High |
| Hyperuricaemia | 57/484 | 36/331 | 1.11 [0.54, 2.28] | Low | Serious (-1) | No indirectness | No imprecision | Unlikely | Medium |
| Blood bilirubin increased | 109/954 | 57/643 | 1.34 [0.81, 2.24] | Low | Serious (-1) | No indirectness | No imprecision | Unlikely | Medium |
| Hepatic function abnormal | 59/525 | 36/366 | 1.45 [0.98, 2.14] | Low | No inconsistency | No indirectness | No imprecision | Unlikely | High |
| Blood creatinine increased | 73/655 | 35/500 | 1.78 [1.21, 2.63] | Low | No inconsistency | No indirectness | No imprecision | Unlikely | High |
| Hypertriglyceridaemia | 55/495 | 33/334 | 1.50 [1.02, 2.23] | Low | No inconsistency | No indirectness | No imprecision | Unlikely | High |
| Pneumonia | 149/1478 | 94/1179 | 1.31 [1.03, 1.66] | Low | No inconsistency | No indirectness | No imprecision | Unlikely | High |
| Lymphocyte count decreased | 79/791 | 44/630 | 1.57 [1.10, 2.23] | Low | No inconsistency | No indirectness | No imprecision | Unlikely | High |
| Hemoptysis | 116/1168 | 89/849 | 1.06 [0.82, 1.37] | Low | No inconsistency | No indirectness | No imprecision | Unlikely | High |
| Edema peripheral | 88/907 | 48/590 | 1.26 [0.69, 2.27] | Low | Serious (-1) | No indirectness | No imprecision | Unlikely | Medium |
| Stomatitis | 34/415 | 17/255 | 1.83 [1.09, 3.06] | Low | No inconsistency | No indirectness | No imprecision | Unlikely | High |
| Upper respiratory tract infection | 32/472 | 10/321 | 2.89 [1.50, 5.56] | Low | No inconsistency | No indirectness | No imprecision | Unlikely | High |
| Hypercholesteraemia | 32/495 | 15/334 | 1.87 [1.03, 3.39] | Low | No inconsistency | No indirectness | No imprecision | Unlikely | High |
| Myelosuppression | 29/525 | 14/366 | 1.37 [0.37, 5.12] | Low | Serious (-1) | No indirectness | No imprecision | Unlikely | Medium |
| Dyspnea | 16/402 | 5/240 | 2.56 [0.98, 6.69] | Low | No inconsistency | No indirectness | No imprecision | Unlikely | High |
| Maculopopular rash | 12/368 | 3/212 | 3.35 [1.05, 10.64] | Low | No inconsistency | No indirectness | No imprecision | Unlikely | High |
| Hypertension | 10/402 | 5/240 | 1.51 [0.54, 4.29] | Low | No inconsistency | No indirectness | No imprecision | Unlikely | High |
| **Grade 3-5** |  |  |  |  |  |  |  |  |  |
| Neutrophil count decreased | 718/1700 | 571/1404 | 1.08 [0.99, 1.17] | Low | No inconsistency | No indirectness | No imprecision | Unlikely | High |
| Neutropenia | 341/1149 | 189/724 | 1.07 [0.94, 1.23] | Low | No inconsistency | No indirectness | No imprecision | Unlikely | High |
| White blood cell decreased | 367/1700 | 291/1404 | 1.10 [0.96, 1.26] | Low | No inconsistency | No indirectness | No imprecision | Unlikely | High |
| Leukopenia | 193/1101 | 106/671 | 1.04 [0.85, 1.27] | Low | No inconsistency | No indirectness | No imprecision | Unlikely | High |
| Anaemia | 375/2232 | 265/1671 | 1.01 [0.88, 1.16] | Low | No inconsistency | No indirectness | No imprecision | Unlikely | High |
| Platelet count decreased | 221/1675 | 158/1389 | 1.18 [0.99, 1.41] | Low | No inconsistency | No indirectness | No imprecision | Unlikely | High |
| Thrombocytopenia | 109/1019 | 54/590 | 1.10 [0.81, 1.49] | Low | No inconsistency | No indirectness | No imprecision | Unlikely | High |
| Pneumonia | 69/1478 | 49/1179 | 1.15 [0.81, 1.62] | Low | No inconsistency | No indirectness | No imprecision | Unlikely | High |
| Lymphocyte count decreased | 34/791 | 11/630 | 2.56 [1.34, 4.87] | Low | No inconsistency | No indirectness | No imprecision | Unlikely | High |
| Myelosuppression | 19/525 | 7/366 | 2.18 [0.96, 4.95] | Low | No inconsistency | No indirectness | No imprecision | Unlikely | High |
| Hyponatraemia | 35/1185 | 21/870 | 1.41 [0.82, 2.41] | Low | No inconsistency | No indirectness | No imprecision | Unlikely | High |
| Dysgeusia | 2/73 | 0/68 | 3.08 [0.16, 60.08] | Low | No inconsistency | No indirectness | No imprecision | Unlikely | High |
| Hypokalaemia | 20/951 | 10/631 | 1.36 [0.66, 2.78] | Low | No inconsistency | No indirectness | No imprecision | Unlikely | High |
| Hepatic function abnormal | 10/525 | 3/366 | 2.18 [0.62, 7.62] | Low | No inconsistency | No indirectness | No imprecision | Unlikely | High |
| Gamma-glutamyltransferase increased | 18/975 | 8/818 | 1.74 [0.77, 3.94] | Low | No inconsistency | No indirectness | No imprecision | Unlikely | High |
| Hypertriglyceridaemia | 9/495 | 3/334 | 2.36 [0.69, 8.04] | Low | No inconsistency | No indirectness | No imprecision | Unlikely | High |
| Myalgia | 2/113 | 1/113 | 1.67 [0.23, 12.14] | Low | No inconsistency | No indirectness | No imprecision | Unlikely | High |
| Alanine aminotransferase increased | 39/2210 | 25/1643 | 1.13 [0.71, 1.78] | Low | No inconsistency | No indirectness | No imprecision | Unlikely | High |
| Diarrhea | 20/1174 | 4/863 | 3.10 [1.20, 7.98] | Low | No inconsistency | No indirectness | No imprecision | Unlikely | High |
| Rash | 23/1743 | 7/1429 | 2.12 [1.04, 4.31] | Low | No inconsistency | No indirectness | No imprecision | Unlikely | High |
| Fatigue | 15/1204 | 11/778 | 0.81 [0.38, 1.73] | Low | No inconsistency | No indirectness | No imprecision | Unlikely | High |
| Asthenia | 17/1365 | 14/1073 | 1.00 [0.51, 1.96] | Low | No inconsistency | No indirectness | No imprecision | Unlikely | High |
| Decreased appetite | 27/2232 | 18/1671 | 1.21 [0.70, 2.08] | Low | No inconsistency | No indirectness | No imprecision | Unlikely | High |
| Hyperglycemia | 8/694 | 2/375 | 1.92 [0.48, 7.79] | Low | No inconsistency | No indirectness | No imprecision | Unlikely | High |
| Aspartate aminotransferase increased | 19/2210 | 12/1643 | 1.16 [0.62, 2.19] | Low | No inconsistency | No indirectness | No imprecision | Unlikely | High |
| Maculopopular rash | 3/368 | 0/212 | 3.17 [0.39, 25.65] | Low | No inconsistency | No indirectness | No imprecision | Unlikely | High |
| Vomiting | 18/2210 | 18/1643 | 0.82 [0.43, 1.56] | Low | No inconsistency | No indirectness | No imprecision | Unlikely | High |
| Hemoptysis | 9/1168 | 8/849 | 0.93 [0.38, 2.26] | Low | No inconsistency | No indirectness | No imprecision | Unlikely | High |
| Hypertension | 3/402 | 2/240 | 1.11 [0.21, 5.80] | Low | No inconsistency | No indirectness | No imprecision | Unlikely | High |
| Stomatitis | 3/415 | 0/255 | 2.83 [0.32, 25.30] | Low | No inconsistency | No indirectness | No imprecision | Unlikely | High |
| Malaise | 4/617 | 4/457 | 0.83 [0.26, 2.59] | Low | No inconsistency | No indirectness | No imprecision | Unlikely | High |
| Arthralgia | 4/623 | 0/633 | 3.60 [0.60, 21.71] | Low | No inconsistency | No indirectness | No imprecision | Unlikely | High |
| Upper respiratory tract infection | 3/472 | 0/321 | 2.98 [0.36, 24.63] | Low | No inconsistency | No indirectness | No imprecision | Unlikely | High |
| Nausea | 14/2232 | 12/1671 | 0.84 [0.42, 1.68] | Low | No inconsistency | No indirectness | No imprecision | Unlikely | High |
| Pain in extremity | 8/1430 | 5/993 | 1.04 [0.43, 2.53] | Low | No inconsistency | No indirectness | No imprecision | Unlikely | High |
| Cough | 3/614 | 1/348 | 1.17 [0.17, 7.88] | Low | No inconsistency | No indirectness | No imprecision | Unlikely | High |
| Blood bilirubin increased | 4/954 | 1/643 | 1.68 [0.36, 7.91] | Low | No inconsistency | No indirectness | No imprecision | Unlikely | High |
| Hypercholesteraemia | 2/495 | 0/334 | 2.14 [0.23, 19.82] | Low | No inconsistency | No indirectness | No imprecision | Unlikely | High |
| Pyrexia | 4/1244 | 1/959 | 1.70 [0.47, 6.08] | Low | No inconsistency | No indirectness | No imprecision | Unlikely | High |
| Blood creatinine increased | 2/655 | 1/500 | 1.20 [0.15, 9.67] | Low | No inconsistency | No indirectness | No imprecision | Unlikely | High |
| Weight decreased | 3/1077 | 5/795 | 0.57 [0.18, 1.80] | Low | No inconsistency | No indirectness | No imprecision | Unlikely | High |
| Dyspnea | 1/402 | 0/240 | 1.50 [0.06, 36.50] | Low | No inconsistency | No indirectness | No imprecision | Unlikely | High |
| Edema peripheral | 2/907 | 0/590 | 1.51 [0.16, 14.43] | Low | No inconsistency | No indirectness | No imprecision | Unlikely | High |
| Alopecia | 2/920 | 1/754 | 1.21 [0.15, 9.70] | Low | No inconsistency | No indirectness | No imprecision | Unlikely | High |
| Hyperuricaemia | 1/484 | 1/331 | 1.00 [0.06, 15.86] | Low | No inconsistency | No indirectness | No imprecision | Unlikely | High |
| Hypothyroidism | 2/1027 | 0/718 | 2.17 [0.23, 20.08] | Low | No inconsistency | No indirectness | No imprecision | Unlikely | High |
| Hypoesthesia | 1/553 | 2/552 | 0.51 [0.05, 5.55] | Low | No inconsistency | No indirectness | No imprecision | Unlikely | High |
| Constipation | 3/2039 | 1/1475 | 1.80 [0.44, 7.34] | Low | No inconsistency | No indirectness | No imprecision | Unlikely | High |
| Hypoalbuminaemia | 2/1560 | 1/994 | 1.40 [0.28, 7.02] | Low | No inconsistency | No indirectness | No imprecision | Unlikely | High |
| **irAEs** |  |  |  |  |  |  |  |  |  |
| **Any grade** |  |  |  |  |  |  |  |  |  |
| Hypothyroidism | 317/1834 | 84/1268 | 4.69 [1.99, 11.03] | Low | Serious (-1) | No indirectness | No imprecision | Unlikely | Medium |
| Severe skin reactions | 74/710 | 15/434 | 3.07 [1.86, 5.08] | Low | No inconsistency | No indirectness | No imprecision | Unlikely | High |
| Aspartate aminotransferase increased | 57/623 | 19/340 | 2.06 [1.27, 3.34] | Low | No inconsistency | No indirectness | No imprecision | Unlikely | High |
| Alanine aminotransferase increased | 57/623 | 21/340 | 1.86 [1.18, 2.94] | Low | No inconsistency | No indirectness | No imprecision | Unlikely | High |
| Pneumonia | 41/484 | 10/331 | 2.15 [1.13, 4.09] | Low | No inconsistency | No indirectness | No imprecision | Unlikely | High |
| Rash | 108/1379 | 48/933 | 2.02 [0.70, 5.82] | Low | Serious (-1) | No indirectness | No imprecision | Unlikely | Medium |
| Pneumonitis | 123/1659 | 26/1093 | 3.12 [2.07, 4.69] | Low | No inconsistency | No indirectness | No imprecision | Unlikely | High |
| Hypokalemia | 29/429 | 16/277 | 1.71 [0.98, 3.00] | Low | No inconsistency | No indirectness | No imprecision | Unlikely | High |
| Hyperthyroidism | 119/1786 | 16/1215 | 4.35 [2.68, 7.06] | Low | No inconsistency | No indirectness | No imprecision | Unlikely | High |
| Hepatitis | 57/1076 | 26/656 | 1.92 [1.31, 2.80] | Low | No inconsistency | No indirectness | No imprecision | Unlikely | High |
| Infusion reactions | 9/194 | 2/184 | 2.87 [0.89, 9.19] | Low | No inconsistency | No indirectness | No imprecision | Unlikely | High |
| Diarrhea | 30/802 | 15/518 | 1.50 [0.83, 2.71] | Low | No inconsistency | No indirectness | No imprecision | Unlikely | High |
| Pyrexia | 18/488 | 6/334 | 2.20 [0.87, 5.60] | Low | No inconsistency | No indirectness | No imprecision | Unlikely | High |
| Blood thyroid-stimulating hormoneincreased | 16/445 | 7/309 | 1.44 [0.57, 3.63] | Low | No inconsistency | No indirectness | No imprecision | Unlikely | High |
| Amylase increased | 19/575 | 12/287 | 0.96 [0.14, 6.66] | Low | Serious (-1) | No indirectness | No imprecision | Unlikely | Medium |
| Adrenal insufficiency | 4/129 | 0/124 | 3.24 [0.54, 19.39] | Low | No inconsistency | No indirectness | No imprecision | Unlikely | High |
| Pruritus | 22/802 | 13/518 | 1.25 [0.64, 2.43] | Low | No inconsistency | No indirectness | No imprecision | Unlikely | High |
| Diabetes | 18/1037 | 4/607 | 1.96 [0.78, 4.93] | Low | No inconsistency | No indirectness | No imprecision | Unlikely | High |
| Gamma-glutamyltransferase increased | 5/357 | 4/209 | 0.90 [0.25, 3.31] | Low | No inconsistency | No indirectness | No imprecision | Unlikely | High |
| Colitis | 9/775 | 1/494 | 2.63 [0.73, 9.54] | Low | No inconsistency | No indirectness | No imprecision | Unlikely | High |
| Thyroiditis | 8/753 | 1/466 | 2.21 [0.63, 7.77] | Low | No inconsistency | No indirectness | No imprecision | Unlikely | High |
| Myocarditis | 9/972 | 0/547 | 3.15 [0.69, 14.34] | Low | No inconsistency | No indirectness | No imprecision | Unlikely | High |
| Nephritis | 5/663 | 0/391 | 2.99 [0.36, 24.75] | Low | No inconsistency | No indirectness | No imprecision | Unlikely | High |
| Pancreatitis | 4/629 | 0/315 | 2.52 [0.30, 21.43] | Low | No inconsistency | No indirectness | No imprecision | Unlikely | High |
| **Grade 3-5** |  |  |  |  |  |  |  |  |  |
| Pneumonitis | 30/1659 | 7/1093 | 2.51 [1.17, 5.35] | Low | No inconsistency | No indirectness | No imprecision | Unlikely | High |
| Severe skin reactions | 12/710 | 0/434 | 4.66 [1.12, 19.43] | Low | No inconsistency | No indirectness | No imprecision | Unlikely | High |
| Hypothyroidism | 27/1659 | 5/1093 | 2.64 [1.07, 6.48] | Low | No inconsistency | No indirectness | No imprecision | Unlikely | High |
| Pneumonia | 7/484 | 0/331 | 5.04 [0.59, 43.25] | Low | No inconsistency | No indirectness | No imprecision | Unlikely | High |
| Hepatitis | 13/1076 | 3/656 | 2.14 [0.75, 6.11] | Low | No inconsistency | No indirectness | No imprecision | Unlikely | High |
| Rash | 13/1204 | 6/758 | 1.33 [0.58, 3.03] | Low | No inconsistency | No indirectness | No imprecision | Unlikely | High |
| Diabetes | 8/1037 | 1/607 | 2.80 [0.64, 12.32] | Low | No inconsistency | No indirectness | No imprecision | Unlikely | High |
| Hypokalemia | 3/429 | 2/277 | 1.51 [0.26, 8.89] | Low | No inconsistency | No indirectness | No imprecision | Unlikely | High |
| Colitis | 5/775 | 1/494 | 2.00 [0.45, 8.87] | Low | No inconsistency | No indirectness | No imprecision | Unlikely | High |
| Amylase increased | 3/575 | 0/287 | 3.46 [0.18, 66.51] | Low | No inconsistency | No indirectness | No imprecision | Unlikely | High |
| Infusion reactions | 1/194 | 1/184 | 0.96 [0.14, 6.61] | Low | No inconsistency | No indirectness | No imprecision | Unlikely | High |
| Myocarditis | 4/972 | 0/547 | 2.51 [0.29, 21.40] | Low | No inconsistency | No indirectness | No imprecision | Unlikely | High |
| Diarrhea | 3/802 | 1/518 | 1.93 [0.29, 12.81] | Low | No inconsistency | No indirectness | No imprecision | Unlikely | High |
| Alanine aminotransferase increased | 2/623 | 2/340 | 0.75 [0.11, 5.10] | Low | No inconsistency | No indirectness | No imprecision | Unlikely | High |
| Pancreatitis | 2/629 | 0/315 | 1.51 [0.16, 14.43] | Low | No inconsistency | No indirectness | No imprecision | Unlikely | High |
| Nephritis | 2/663 | 0/391 | 1.50 [0.16, 14.33] | Low | No inconsistency | No indirectness | No imprecision | Unlikely | High |
| Gamma-glutamyltransferase increased | 1/357 | 1/209 | 0.73 [0.09, 6.10] | Low | No inconsistency | No indirectness | No imprecision | Unlikely | High |
| Thyroiditis | 1/753 | 0/466 | 3.02 [0.12, 73.52] | Low | No inconsistency | No indirectness | No imprecision | Unlikely | High |
| Aspartate aminotransferase increased | 0/623 | 2/340 | 0.22 [0.01, 4.48] | Low | No inconsistency | No indirectness | No imprecision | Unlikely | High |

**Abbreviations:** AE: Adverse event; ALT: Alanine aminotransferase; AST: Aspartate aminotransferase; CPS: Combined positive score; CR: Complete response; CI: confidence interval; DCR: Disease control rate; DOR: Duration of response; DORR: Duration of response rate; ECOG PS: Eastern Cooperative Oncology Group Performance Status; GRADE: Grading of Recommendations, Assessment, Development, and Evaluation; HR: Hazard ratio; irAE: Immune-related adverse event; NSCLC: Non-small-cell lung cancer; ORR: Objective response rate; OS: Overall survival; OSR: Overall survival rate; P: Probability; PC: PD-1/PD-L1 inhibitors combined with chemotherapy; PD: Progressive disease; PD-1: Programmed death-1; PD-L1: Programmed death-ligand 1; PFS: Progression-free survival; PFSR: Progression-free survival rate; PR: Partial response; RCT: Randomized controlled trial; RR: Risk ratio; SD: Stable disease; TEAE: Treatment-emergent adverse event; TRAEs: Treatment-related adverse events.

^a^ Differences: HR for OS, PFS and DOR; RR for OSR, PFSR, DORR, responses, and AEs.

^b^ Risk of bias assessed using the Jadad scale for randomized controlled trials.

^c^ Publication bias was explored through visual inspection of the funnel plots.
