# Supplementary material for: First-line immunochemotherapy for advanced NSCLC in Asian patients: a meta-analysis of phase 3 RCTs
Source: Front Oncol. 2025 Nov 19;15:1709348. doi: 10.3389/fonc.2025.1709348 (PMC12672283; doi:10.3389/fonc.2025.1709348)
Supplement: Supplementary file 12 [file Table4.doc]

**Table S4** Any grade treatment-emergent adverse events.

| **TEAEs** | **PC** | | **Chemotherapy** | | **Risk ratio [95% CI]** | **P** |
| --- | --- | --- | --- | --- | --- | --- |
|  | **Event/total** | **%** | **Event/total** | **%** |  |  |
| Anaemia | 1700/2232 | 76.16% | 1218/1671 | 72.89% | 1.02 [0.99, 1.06] | 0.23 |
| Neutrophil count decreased | 1166/1700 | 68.59% | 918/1404 | 65.38% | 1.06 [1.01, 1.12] | 0.01 |
| White blood cell decreased | 1138/1700 | 66.94% | 903/1404 | 64.32% | 1.06 [1.00, 1.11] | 0.03 |
| Alopecia | 463/920 | 50.33% | 411/754 | 54.51% | 1.05 [0.97, 1.14] | 0.19 |
| Leukopenia | 549/1101 | 49.86% | 299/671 | 44.56% | 1.04 [0.96, 1.13] | 0.29 |
| Neutropenia | 554/1149 | 48.22% | 314/724 | 43.37% | 1.05 [0.90, 1.22] | 0.57 |
| Thrombocytopenia | 434/1019 | 42.59% | 234/590 | 39.66% | 1.02 [0.92, 1.13] | 0.67 |
| Platelet count decreased | 682/1675 | 40.72% | 530/1389 | 38.16% | 1.08 [0.99, 1.18] | 0.07 |
| ALT increased | 821/2210 | 37.15% | 494/1643 | 30.07% | 1.19 [1.09, 1.31] | 0.0002 |
| AST increased | 781/2210 | 35.34% | 410/1643 | 24.95% | 1.38 [1.16, 1.63] | 0.0002 |
| Nausea | 776/2232 | 34.77% | 534/1671 | 31.96% | 1.08 [0.98, 1.18] | 0.11 |
| Hypoesthesia | 191/553 | 34.54% | 160/552 | 28.99% | 1.19 [1.00, 1.41] | 0.05 |
| Decreased appetite | 745/2232 | 33.38% | 496/1671 | 29.68% | 1.13 [1.02, 1.24] | 0.01 |
| Asthenia | 371/1365 | 27.18% | 272/1073 | 25.35% | 1.10 [0.96, 1.26] | 0.16 |
| Constipation | 499/2039 | 24.47% | 357/1475 | 24.20% | 1.05 [0.94, 1.18] | 0.41 |
| Fatigue | 284/1204 | 23.59% | 136/778 | 17.48% | 1.24 [0.86, 1.80] | 0.25 |
| Pyrexia | 270/1244 | 21.70% | 154/959 | 16.06% | 1.36 [1.14, 1.63] | 0.0007 |
| Vomiting | 459/2210 | 20.77% | 326/1643 | 19.84% | 1.03 [0.91, 1.17] | 0.64 |
| Hypoalbuminaemia | 300/1560 | 19.23% | 151/994 | 15.19% | 1.27 [1.06, 1.52] | 0.01 |
| Cough | 113/614 | 18.40% | 49/348 | 14.08% | 1.26 [0.92, 1.71] | 0.15 |
| Rash | 318/1743 | 18.24% | 118/1429 | 8.26% | 2.13 [1.75, 2.59] | < 0.00001 |
| Pain in extremity | 255/1430 | 17.83% | 180/993 | 18.13% | 1.07 [0.90, 1.27] | 0.43 |
| Arthralgia | 105/623 | 16.85% | 91/633 | 14.38% | 1.17 [0.91, 1.51] | 0.22 |
| Myalgia | 18/113 | 15.93% | 9/113 | 7.96% | 1.91 [0.91, 4.01] | 0.09 |
| Dysgeusia | 11/73 | 15.07% | 10/68 | 14.71% | 0.83 [0.18, 3.96] | 0.82 |
| Malaise | 88/617 | 14.26% | 71/457 | 15.54% | 1.12 [0.85, 1.49] | 0.42 |
| Gamma-glutamyltransferase increased | 136/975 | 13.95% | 93/818 | 11.37% | 1.22 [0.83, 1.81] | 0.32 |
| Weight decreased | 149/1077 | 13.83% | 112/795 | 14.09% | 1.01 [0.67, 1.54] | 0.95 |
| Diarrhea | 154/1174 | 13.12% | 86/863 | 9.97% | 1.41 [1.10, 1.81] | 0.007 |
| Hypothyroidism | 133/1027 | 12.95% | 15/718 | 2.09% | 6.01 [3.56, 10.12] | < 0.00001 |
| Hyperglycemia | 89/694 | 12.82% | 34/375 | 9.07% | 1.47 [1.01, 2.12] | 0.04 |
| Hyponatraemia | 147/1185 | 12.41% | 103/870 | 11.84% | 1.18 [0.93, 1.50] | 0.17 |
| Hypokalaemia | 117/951 | 12.30% | 71/631 | 11.25% | 1.13 [0.86, 1.48] | 0.38 |
| Hyperthyroidism | 47/391 | 12.02% | 4/237 | 1.69% | 6.96 [2.55, 18.98] | 0.0001 |
| Hyperuricaemia | 57/484 | 11.78% | 36/331 | 10.88% | 1.11 [0.54, 2.28] | 0.77 |
| Blood bilirubin increased | 109/954 | 11.43% | 57/643 | 8.86% | 1.34 [0.81, 2.24] | 0.26 |
| Hepatic function abnormal | 59/525 | 11.24% | 36/366 | 9.84% | 1.45 [0.98, 2.14] | 0.06 |
| Blood creatinine increased | 73/655 | 11.15% | 35/500 | 7.00% | 1.78 [1.21, 2.63] | 0.004 |
| Hypertriglyceridaemia | 55/495 | 11.11% | 33/334 | 9.88% | 1.50 [1.02, 2.23] | 0.04 |
| Pneumonia | 149/1478 | 10.08% | 94/1179 | 7.97% | 1.31 [1.03, 1.66] | 0.03 |
| Lymphocyte count decreased | 79/791 | 9.99% | 44/630 | 6.98% | 1.57 [1.10, 2.23] | 0.01 |
| Hemoptysis | 116/1168 | 9.93% | 89/849 | 10.48% | 1.06 [0.82, 1.37] | 0.68 |
| Edema peripheral | 88/907 | 9.70% | 48/590 | 8.14% | 1.26 [0.69, 2.27] | 0.45 |
| Stomatitis | 34/415 | 8.19% | 17/255 | 6.67% | 1.83 [1.09, 3.06] | 0.02 |
| Upper respiratory tract infection | 32/472 | 6.78% | 10/321 | 3.12% | 2.89 [1.50, 5.56] | 0.001 |
| Hypercholesteraemia | 32/495 | 6.46% | 15/334 | 4.49% | 1.87 [1.03, 3.39] | 0.04 |
| Myelosuppression | 29/525 | 5.52% | 14/366 | 3.83% | 1.37 [0.37, 5.12] | 0.64 |
| Dyspnea | 16/402 | 3.98% | 5/240 | 2.08% | 2.56 [0.98, 6.69] | 0.05 |
| Maculopopular rash | 12/368 | 3.26% | 3/212 | 1.42% | 3.35 [1.05, 10.64] | 0.04 |
| Hypertension | 10/402 | 2.49% | 5/240 | 2.08% | 1.51 [0.54, 4.29] | 0.43 |

**Abbreviations:** AE: Adverse event; ALT: Alanine aminotransferase; AST: Aspartate aminotransferase; CI: Confidence interval; PC: PD-1/PD-L1 inhibitors combined with chemotherapy; PD-1: Programmed cell death protein 1; PD-L1: Programmed death-ligand 1; RR: Risk ratio; TEAE: Treatment-emergent adverse event.
