# Supplementary material for: First-line immunochemotherapy for advanced NSCLC in Asian patients: a meta-analysis of phase 3 RCTs
Source: Front Oncol. 2025 Nov 19;15:1709348. doi: 10.3389/fonc.2025.1709348 (PMC12672283; doi:10.3389/fonc.2025.1709348)
Supplement: Supplementary file 13 [file Table5.doc]

**Table S5** Grade 3-5 treatment-emergent adverse events.

| **Grade 3-5 TEAEs** | **PC** | | **Chemotherapy** | | **Risk ratio [95% CI]** | **P** |
| --- | --- | --- | --- | --- | --- | --- |
|  | **Event/total** | **%** | **Event/total** | **%** |  |  |
| Neutrophil count decreased | 718/1700 | 42.24% | 571/1404 | 40.67% | 1.08 [0.99, 1.17] | 0.07 |
| Neutropenia | 341/1149 | 29.68% | 189/724 | 26.10% | 1.07 [0.94, 1.23] | 0.31 |
| White blood cell decreased | 367/1700 | 21.59% | 291/1404 | 20.73% | 1.10 [0.96, 1.26] | 0.15 |
| Leukopenia | 193/1101 | 17.53% | 106/671 | 15.80% | 1.04 [0.85, 1.27] | 0.71 |
| Anaemia | 375/2232 | 16.80% | 265/1671 | 15.86% | 1.01 [0.88, 1.16] | 0.88 |
| Platelet count decreased | 221/1675 | 13.19% | 158/1389 | 11.38% | 1.18 [0.99, 1.41] | 0.07 |
| Thrombocytopenia | 109/1019 | 10.70% | 54/590 | 9.15% | 1.10 [0.81, 1.49] | 0.53 |
| Pneumonia | 69/1478 | 4.67% | 49/1179 | 4.16% | 1.15 [0.81, 1.62] | 0.44 |
| Lymphocyte count decreased | 34/791 | 4.30% | 11/630 | 1.75% | 2.56 [1.34, 4.87] | 0.004 |
| Myelosuppression | 19/525 | 3.62% | 7/366 | 1.91% | 2.18 [0.96, 4.95] | 0.06 |
| Hyponatraemia | 35/1185 | 2.95% | 21/870 | 2.41% | 1.41 [0.82, 2.41] | 0.22 |
| Dysgeusia | 2/73 | 2.74% | 0/68 | 0.00% | 3.08 [0.16, 60.08] | 0.46 |
| Hypokalaemia | 20/951 | 2.10% | 10/631 | 1.58% | 1.36 [0.66, 2.78] | 0.40 |
| Hepatic function abnormal | 10/525 | 1.90% | 3/366 | 0.82% | 2.18 [0.62, 7.62] | 0.22 |
| Gamma-glutamyltransferase increased | 18/975 | 1.85% | 8/818 | 0.98% | 1.74 [0.77, 3.94] | 0.18 |
| Hypertriglyceridaemia | 9/495 | 1.82% | 3/334 | 0.90% | 2.36 [0.69, 8.04] | 0.17 |
| Myalgia | 2/113 | 1.77% | 1/113 | 0.88% | 1.67 [0.23, 12.14] | 0.61 |
| ALT increased | 39/2210 | 1.76% | 25/1643 | 1.52% | 1.13 [0.71, 1.78] | 0.61 |
| Diarrhea | 20/1174 | 1.70% | 4/863 | 0.46% | 3.10 [1.20, 7.98] | 0.02 |
| Rash | 23/1743 | 1.32% | 7/1429 | 0.49% | 2.12 [1.04, 4.31] | 0.04 |
| Fatigue | 15/1204 | 1.25% | 11/778 | 1.41% | 0.81 [0.38, 1.73] | 0.59 |
| Asthenia | 17/1365 | 1.25% | 14/1073 | 1.30% | 1.00 [0.51, 1.96] | 0.99 |
| Decreased appetite | 27/2232 | 1.21% | 18/1671 | 1.08% | 1.21 [0.70, 2.08] | 0.49 |
| Hyperglycemia | 8/694 | 1.15% | 2/375 | 0.53% | 1.92 [0.48, 7.79] | 0.36 |
| AST increased | 19/2210 | 0.86% | 12/1643 | 0.73% | 1.16 [0.62, 2.19] | 0.65 |
| Maculopopular rash | 3/368 | 0.82% | 0/212 | 0.00% | 3.17 [0.39, 25.65] | 0.28 |
| Vomiting | 18/2210 | 0.81% | 18/1643 | 1.10% | 0.82 [0.43, 1.56] | 0.55 |
| Hemoptysis | 9/1168 | 0.77% | 8/849 | 0.94% | 0.93 [0.38, 2.26] | 0.87 |
| Hypertension | 3/402 | 0.75% | 2/240 | 0.83% | 1.11 [0.21, 5.80] | 0.90 |
| Stomatitis | 3/415 | 0.72% | 0/255 | 0.00% | 2.83 [0.32, 25.30] | 0.35 |
| Malaise | 4/617 | 0.65% | 4/457 | 0.88% | 0.83 [0.26, 2.59] | 0.75 |
| Arthralgia | 4/623 | 0.64% | 0/633 | 0.00% | 3.60 [0.60, 21.71] | 0.16 |
| Upper respiratory tract infection | 3/472 | 0.64% | 0/321 | 0.00% | 2.98 [0.36, 24.63] | 0.31 |
| Nausea | 14/2232 | 0.63% | 12/1671 | 0.72% | 0.84 [0.42, 1.68] | 0.62 |
| Pain in extremity | 8/1430 | 0.56% | 5/993 | 0.50% | 1.04 [0.43, 2.53] | 0.93 |
| Cough | 3/614 | 0.49% | 1/348 | 0.29% | 1.17 [0.17, 7.88] | 0.87 |
| Blood bilirubin increased | 4/954 | 0.42% | 1/643 | 0.16% | 1.68 [0.36, 7.91] | 0.51 |
| Hypercholesteraemia | 2/495 | 0.40% | 0/334 | 0.00% | 2.14 [0.23, 19.82] | 0.50 |
| Pyrexia | 4/1244 | 0.32% | 1/959 | 0.10% | 1.70 [0.47, 6.08] | 0.42 |
| Blood creatinine increased | 2/655 | 0.31% | 1/500 | 0.20% | 1.20 [0.15, 9.67] | 0.86 |
| Weight decreased | 3/1077 | 0.28% | 5/795 | 0.63% | 0.57 [0.18, 1.80] | 0.34 |
| Dyspnea | 1/402 | 0.25% | 0/240 | 0.00% | 1.50 [0.06, 36.50] | 0.81 |
| Edema peripheral | 2/907 | 0.22% | 0/590 | 0.00% | 1.51 [0.16, 14.43] | 0.72 |
| Alopecia | 2/920 | 0.22% | 1/754 | 0.13% | 1.21 [0.15, 9.70] | 0.86 |
| Hyperuricaemia | 1/484 | 0.21% | 1/331 | 0.30% | 1.00 [0.06, 15.86] | 1.00 |
| Hypothyroidism | 2/1027 | 0.19% | 0/718 | 0.00% | 2.17 [0.23, 20.08] | 0.50 |
| Hypoesthesia | 1/553 | 0.18% | 2/552 | 0.36% | 0.51 [0.05, 5.55] | 0.58 |
| Constipation | 3/2039 | 0.15% | 1/1475 | 0.07% | 1.80 [0.44, 7.34] | 0.41 |
| Hypoalbuminaemia | 2/1560 | 0.13% | 1/994 | 0.10% | 1.40 [0.28, 7.02] | 0.68 |

**Abbreviations:** AE: Adverse event; ALT: Alanine aminotransferase; AST: Aspartate aminotransferase; CI: Confidence interval; PC: PD-1/PD-L1 inhibitors combined with chemotherapy; PD-1: Programmed cell death protein 1; PD-L1: Programmed death-ligand 1; RR: Risk ratio; TEAE: Treatment-emergent adverse event.
