# Supplementary material for: First-line immunochemotherapy for advanced NSCLC in Asian patients: a meta-analysis of phase 3 RCTs
Source: Front Oncol. 2025 Nov 19;15:1709348. doi: 10.3389/fonc.2025.1709348 (PMC12672283; doi:10.3389/fonc.2025.1709348)
Supplement: Supplementary file 14 [file Table6.doc]

**Table S6** Any grade immune-related adverse events.

| **irAEs** | **PC** | | **Chemotherapy** | | **Risk ratio [95% CI]** | **P** |
| --- | --- | --- | --- | --- | --- | --- |
|  | **Event/total** | **%** | **Event/total** | **%** |  |  |
| Hypothyroidism | 317/1834 | 17.28% | 84/1268 | 6.62% | 4.69 [1.99, 11.03] | 0.0004 |
| Severe skin reactions | 74/710 | 10.42% | 15/434 | 3.46% | 3.07 [1.86, 5.08] | < 0.0001 |
| AST increased | 57/623 | 9.15% | 19/340 | 5.59% | 2.06 [1.27, 3.34] | 0.003 |
| ALT increased | 57/623 | 9.15% | 21/340 | 6.18% | 1.86 [1.18, 2.94] | 0.008 |
| Pneumonia | 41/484 | 8.47% | 10/331 | 3.02% | 2.15 [1.13, 4.09] | 0.02 |
| Rash | 108/1379 | 7.83% | 48/933 | 5.14% | 2.02 [0.70, 5.82] | 0.19 |
| Pneumonitis | 123/1659 | 7.41% | 26/1093 | 2.38% | 3.12 [2.07, 4.69] | < 0.00001 |
| Hypokalemia | 29/429 | 6.76% | 16/277 | 5.78% | 1.71 [0.98, 3.00] | 0.06 |
| Hyperthyroidism | 119/1786 | 6.66% | 16/1215 | 1.32% | 4.35 [2.68, 7.06] | < 0.00001 |
| Hepatitis | 57/1076 | 5.30% | 26/656 | 3.96% | 1.92 [1.31, 2.80] | 0.0008 |
| Infusion reactions | 9/194 | 4.64% | 2/184 | 1.09% | 2.87 [0.89, 9.19] | 0.08 |
| Diarrhea | 30/802 | 3.74% | 15/518 | 2.90% | 1.50 [0.83, 2.71] | 0.18 |
| Pyrexia | 18/488 | 3.69% | 6/334 | 1.80% | 2.20 [0.87, 5.60] | 0.10 |
| Blood thyroid-stimulating hormoneincreased | 16/445 | 3.60% | 7/309 | 2.27% | 1.44 [0.57, 3.63] | 0.44 |
| Amylase increased | 19/575 | 3.30% | 12/287 | 4.18% | 0.96 [0.14, 6.66] | 0.96 |
| Adrenal insufficiency | 4/129 | 3.10% | 0/124 | 0.00% | 3.24 [0.54, 19.39] | 0.20 |
| Pruritus | 22/802 | 2.74% | 13/518 | 2.51% | 1.25 [0.64, 2.43] | 0.52 |
| Diabetes | 18/1037 | 1.74% | 4/607 | 0.66% | 1.96 [0.78, 4.93] | 0.15 |
| Gamma-glutamyltransferase increased | 5/357 | 1.40% | 4/209 | 1.91% | 0.90 [0.25, 3.31] | 0.88 |
| Colitis | 9/775 | 1.16% | 1/494 | 0.20% | 2.63 [0.73, 9.54] | 0.14 |
| Thyroiditis | 8/753 | 1.06% | 1/466 | 0.21% | 2.21 [0.63, 7.77] | 0.22 |
| Myocarditis | 9/972 | 0.93% | 0/547 | 0.00% | 3.15 [0.69, 14.34] | 0.14 |
| Nephritis | 5/663 | 0.75% | 0/391 | 0.00% | 2.99 [0.36, 24.75] | 0.31 |
| Pancreatitis | 4/629 | 0.64% | 0/315 | 0.00% | 2.52 [0.30, 21.43] | 0.40 |

**Abbreviations:** AE: Adverse event; ALT: Alanine aminotransferase; AST: Aspartate aminotransferase; CI: Confidence interval; irAE: Immune-related adverse event; PC: PD-1/PD-L1 inhibitors combined with chemotherapy; PD-1: Programmed cell death protein 1; PD-L1: Programmed death-ligand 1; RR: Risk ratio.
