# Supplementary material for: First-line immunochemotherapy for advanced NSCLC in Asian patients: a meta-analysis of phase 3 RCTs
Source: Front Oncol. 2025 Nov 19;15:1709348. doi: 10.3389/fonc.2025.1709348 (PMC12672283; doi:10.3389/fonc.2025.1709348)
Supplement: Supplementary file 15 [file Table7.doc]

**Table S7** Grade 3-5 immune-related adverse events.

| **Grade 3-5 irAEs** | **PC** | | **Chemotherapy** | | **Risk ratio [95% CI]** | **P** |
| --- | --- | --- | --- | --- | --- | --- |
|  | **Event/total** | **%** | **Event/total** | **%** |  |  |
| Pneumonitis | 30/1659 | 1.81% | 7/1093 | 0.64% | 2.51 [1.17, 5.35] | 0.02 |
| Severe skin reactions | 12/710 | 1.69% | 0/434 | 0.00% | 4.66 [1.12, 19.43] | 0.03 |
| Hypothyroidism | 27/1659 | 1.63% | 5/1093 | 0.46% | 2.64 [1.07, 6.48] | 0.03 |
| Pneumonia | 7/484 | 1.45% | 0/331 | 0.00% | 5.04 [0.59, 43.25] | 0.14 |
| Hepatitis | 13/1076 | 1.21% | 3/656 | 0.46% | 2.14 [0.75, 6.11] | 0.16 |
| Rash | 13/1204 | 1.08% | 6/758 | 0.79% | 1.33 [0.58, 3.03] | 0.50 |
| Diabetes | 8/1037 | 0.77% | 1/607 | 0.16% | 2.80 [0.64, 12.32] | 0.17 |
| Hypokalemia | 3/429 | 0.70% | 2/277 | 0.72% | 1.51 [0.26, 8.89] | 0.65 |
| Colitis | 5/775 | 0.65% | 1/494 | 0.20% | 2.00 [0.45, 8.87] | 0.36 |
| Amylase increased | 3/575 | 0.52% | 0/287 | 0.00% | 3.46 [0.18, 66.51] | 0.41 |
| Infusion reactions | 1/194 | 0.52% | 1/184 | 0.54% | 0.96 [0.14, 6.61] | 0.96 |
| Myocarditis | 4/972 | 0.41% | 0/547 | 0.00% | 2.51 [0.29, 21.40] | 0.40 |
| Diarrhea | 3/802 | 0.37% | 1/518 | 0.19% | 1.93 [0.29, 12.81] | 0.50 |
| ALT increased | 2/623 | 0.32% | 2/340 | 0.59% | 0.75 [0.11, 5.10] | 0.77 |
| Pancreatitis | 2/629 | 0.32% | 0/315 | 0.00% | 1.51 [0.16, 14.43] | 0.72 |
| Nephritis | 2/663 | 0.30% | 0/391 | 0.00% | 1.50 [0.16, 14.33] | 0.73 |
| Gamma-glutamyltransferase increased | 1/357 | 0.28% | 1/209 | 0.48% | 0.73 [0.09, 6.10] | 0.77 |
| Thyroiditis | 1/753 | 0.13% | 0/466 | 0.00% | 3.02 [0.12, 73.52] | 0.50 |
| AST increased | 0/623 | 0.00% | 2/340 | 0.59% | 0.22 [0.01, 4.48] | 0.33 |

**Abbreviations:** AE: Adverse event; ALT: Alanine aminotransferase; AST: Aspartate aminotransferase; CI: Confidence interval; irAE: Immune-related adverse event; PC: PD-1/PD-L1 inhibitors combined with chemotherapy; PD-1: Programmed cell death protein 1; PD-L1: Programmed death-ligand 1; RR: Risk ratio.
